# Highlights in gibberellin research: A tale of the dwarf and the slender

**DOI:** 10.1093/plphys/kiae044

**Published:** 2024-01-30

**Authors:** Eilon Shani, Peter Hedden, Tai-ping Sun

**Affiliations:** School of Plant Sciences and Food Security, Tel Aviv University, Tel Aviv 69978, Israel; Laboratory of Growth Regulators, Institute of Experimental Botany and Palacky University, 78371 Olomouc, Czech Republic; Sustainable Soils and Crops, Rothamsted Research, Harpenden AL5 2JQ, UK; Department of Biology, Duke University, Durham, NC 27708, USA

## Abstract

It has been almost a century since biologically active gibberellin (GA) was isolated. Here, we give a historical overview of the early efforts in establishing the GA biosynthesis and catabolism pathway, characterizing the enzymes for GA metabolism, and elucidating their corresponding genes. We then highlight more recent studies that have identified the GA receptors and early GA signaling components (DELLA repressors and F-box activators), determined the molecular mechanism of DELLA-mediated transcription reprograming, and revealed how DELLAs integrate multiple signaling pathways to regulate plant vegetative and reproductive development in response to internal and external cues. Finally, we discuss the GA transporters and their roles in GA-mediated plant development.

## Teaser

A historical overview of gibberellin research showcases important advances in our understanding of gibberellin metabolism, perception, signaling, and transport.

## Introduction

**Figure 1.**
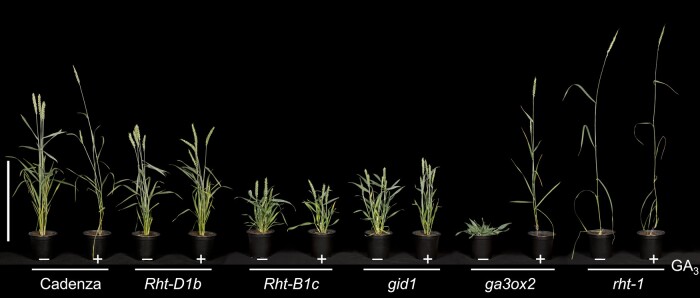
GA regulates plant growth and development. Shown are 12-wk-old GA-unresponsive vs -responsive mutants in wheat. All the mutant lines are in cv. Cadenza background. The gain-of-function *Rht-D1b* and *Rht-B1c* (*DELLA*) alleles were introduced into Cadenza from cvs Avalon and Mercia, respectively (Van De Velde et al. 2021). The hypomorphic *gid1* and loss-of-function *ga3ox2* and *rht-1* plants were produced by TILLING after EMS-induced mutagenesis (A.L. Phillips and S.G. Thomas, unpublished data). All plants were untreated (–) or treated (+) twice-weekly with 10 *µ*M GA_3_. *Rht* and *gid1* mutants are unresponsive to GA treatment, whereas GA completely rescued the GA-biosynthesis mutant *ga3ox2*. Scale bar = 40 cm.

**Figure 2.**
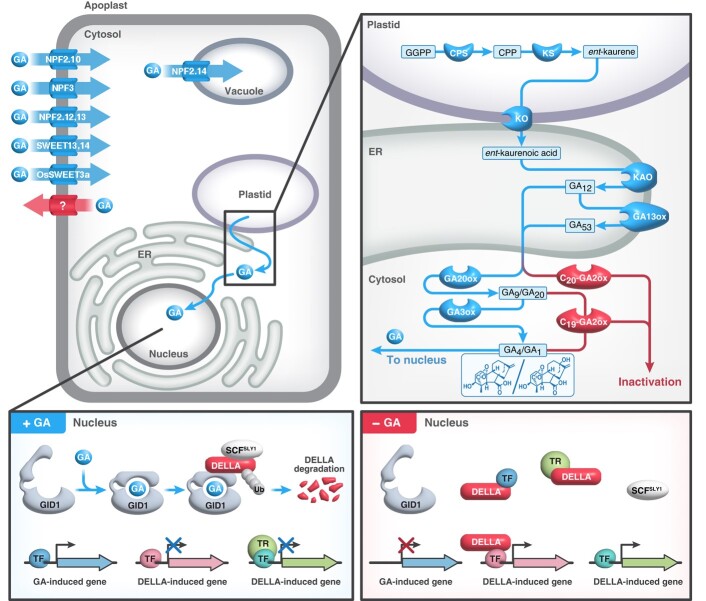
GA metabolism, transport, perception, and signaling in plant cells. GA biosynthesis takes place in three cellular compartments: *ent*-Kaurene is synthesized from GGPP by CPS and KS in the plastid; *ent*-Kaurene is converted to *ent*-kaurenoic acid by KO on the outer plastid membrane, which is connected to the ER; *ent*-Kaurenoic acid is converted to GA_12_ by KAO and GA_12_ to GA_53_ by GA13ox in the ER; GA_12_ and GA_53_ are converted to bioactive GA_4_ and GA_1_, respectively, by GA20ox and GA3ox in the cytoplasm. GA_4_ and GA_1_ as well as their immediate precursors GA_9_ and GA_20_, respectively, are oxidized on C-2 by C_19_-GA2ox, resulting in inactivation, while C_20_-GA2ox acts on earlier C_20_-GA precursors. In addition to de novo biosynthesis, GA can be imported into the cell by GA transporters nitrate and peptide transporter families (NPFs) and SWEETs or transported into the vacuole as labeled. GA perception and signaling occur in the nucleus where GA binding to its receptor GID1 (+GA) promotes DELLA degradation via Skp, Cullin, F-box (SCF)^SLY1/GID2^-mediated polyubiquitination and subsequent proteolysis by the 26S proteasome. When GA levels are low (− GA), DELLAs accumulate to high levels. Three distinct modes of DELLA action are shown: (i) DELLA represses transcription by blocking DNA binding and sequestering transcription factors (TF in blue) from target promoters; (ii) DELLA induces transcription by recruiting TFs (in pink); and (iii) DELLA induces transcription by sequestering transcription repressors (TR in green) from target promoters. GGPP, *trans*-geranylgeranyl diphosphate; CPP, *ent*-copalyl diphosphate; CPS, *ent*-copalyl diphosphate synthase; KS, *ent*-kaurene synthase; KO, *ent*-kaurene oxidase; KAO, *ent*-kaurenoic acid oxidase; GA13ox, GA 13-oxidase; GA20ox, GA 20-oxidase; GA3ox, GA 3-oxidase; GA2ox, GA 2-oxidase. GA biosynthesis enzymes are labeled in blue, and the deactivation enzymes are labeled in red. GA, gibberellin; TF, transcription factor; TR, transcription repressors; ER, endoplasmic reticulum.

The gibberellins (GAs) were named for the phytopathogenic fungus *Gibberella fujikuroi*, whose secretions caused abnormal growth and sterility in infected rice (*Oryza sativa*) plants (Kurosawa 1926). The biologically active factor was isolated in impure form from fungal cultures in the 1930s in Japan and named gibberellin A (Yabuta and Sumiki 1938), but this research was not widely known outside of Japan until the late 1940s. The main active component was identified in the 1950s in the UK, USA, and Japan, where it was named gibberellic acid, gibberellin-X, and gibberellin A_3_ (GA_3_), respectively (Curtis and Cross 1954; Stodola et al. 1955; Takahashi et al. 1955). The structure of gibberellic acid, the name agreed upon by the UK and US groups, or GA_3_, was proposed in the late 1950s by chemists working at the ICI Ackers Laboratory, Welwyn, UK (reviewed by Grove 1961). The remarkable effect of this substance on plants stimulated interest in fungal gibberellins (GAs). GAs promote plant growth, particularly to rescue the growth of dwarf mutants of pea (*Pisum sativum*) and maize (*Zea mays*) (Brian et al. 1954; Phinney 1956) and to induce bolting in long-day (LD) rosette species (Lang 1956; Wittwer et al. 1957), prompting the suggestion that they may be endogenous plant hormones (Stowe and Yamaki 1957). Plant extracts promoted the growth of mutants in a similar manner to GA_3,_ reinforcing this hypothesis, which was confirmed by the isolation of 2 mg of GA_1_ from 87.3 kg of immature seeds of runner bean (*Phaseolus coccineus*) (Macmillan and Suter 1958). The identity of the isolated bioactive compound was determined by comparing its infra-red spectrum with that of authentic GA_1_ from *G. fujikuroi.* Thus, GA was established as the second endogenous growth regulator (plant hormone) after auxin. The role of GA in plant growth regulation is illustrated in Fig. 1, which compares wild-type and mutant wheat (*Triticum aestivum*) plants with compromised GA-biosynthesis (GA-responsive) or signaling (GA-unresponsive) without or with treatment with GA_3_. While GA_3_ was the first GA to be discovered, it is a minor form in plants, whereas the major bioactive forms are GA_1_ or GA_4_ (see Fig. 2). GA_3_ differs from GA_1_ by the presence of a double bond that prevents inactivation by 2β-hydroxylation.

**Advances**

- The use of biosensors, gene reporters, single-cell RNA-sequencing, and tissue-specific manipulation of GA metabolism is revealing the cellular distribution of GA biosynthesis and accumulation, and its relevance in plant development.
- The GA signaling repressors DELLAs function as master growth regulators by interacting with regulators in many cellular pathways in response to internal and external cues.
- Besides GA-GID1-induced degradation, DELLA activity is regulated by interacting transcription factors, GA-GID1-independent polyubiquitination and degradation, and other PTMs (glycosylation, SUMOylation, and phosphorylation).
- The movement of GA precursors provides an additional layer of regulation for bioactive GA contents at responding tissues, which is particularly crucial for long-distance communication in coordinating plant growth and development.

The identification of GA_1_ and other GAs in bean seeds was followed by the isolation of other GAs from many plant species. Their structures were determined by conversion to compounds of known structure and/or nuclear magnetic resonance spectroscopy profiles. Later, the development of combined gas chromatography-mass spectrometry for GA analysis allowed them to be identified in plant tissues without the need to obtain pure compounds (Binks et al. 1969). This technique, and later liquid chromatography–mass spectrometry (LC–MS), has enabled GA identification and quantitation to become routine and not just the purview of chemists. The high sensitivity and resolution of ultra high performance LC-MS-MS now allow GAs to be measured in low (mg) amounts of plant tissues.

Following their discovery, there was initially steady but slow progress in elucidating the biosynthetic pathways for GAs in *G. fujikuroi* (the rice pathogen is now reclassified as *Fusarium fujikuroi*) and in plants. In addition, although there was considerable information on the physiological action of GAs on plants, advances in understanding their molecular modes of action were sluggish. By contrast, the application of GAs in agriculture and horticulture developed rapidly with their availability from fungal cultures, with major uses in the production of seedless grapes, to improve skin finish in apples, and many other applications (Rademacher 2015). Furthermore, inhibitors of GA biosynthesis found important applications as plant growth retardants (Rademacher 2000), while semidwarf varieties of major crop species that were key contributors to the Green Revolution were later shown to be defective in GA biosynthesis or action (Phillips 2016). In the last 30 years, with the use of mutants and developments in molecular genetics and genomics, progress in our understanding of both GA metabolism and signaling has accelerated, with details emerging on the biosynthetic reactions, enzymes, genes, and their regulation as well as GA perception and signal transduction. The movement of GAs between cells or over longer distances between organs is an important factor in their function, and recent progress in understanding their transport and transporters is a major development. This review highlights the advances that have contributed to our current understanding of GA metabolism, signaling, and transport and their role in plant development.

## GA metabolism

### Establishing the GA-biosynthetic pathways

The diterpenoid nature of GAs was demonstrated in the fungus *F. fujikuroi* by the incorporation of ^14^C-labeled mevalonic acid (MVA) into GA_3_ (Birch et al. 1958). Subsequently, the GA-biosynthetic and catabolic pathways were established in plants, primarily using cell-free systems from developing seeds (Graebe et al. 1965; Dennis and West 1967; Graebe et al. 1972, 1974a, b; Kamiya and Graebe 1983) and in the fungus using liquid cultures (Cross et al. 1964; Bearder et al. 1975; Evans and Hanson 1975). The GA metabolism pathway in plants is summarized in Fig. 2, and details can be found in a recent review (Hedden 2020). The diterpene precursor *trans*-geranylgeranyl diphosphate (GGPP), which is formed from MVA in the fungus and mainly via the methylerythritol phosphate (MEP) pathway in plants, is converted in two steps to the tetracyclic diterpene *ent*-kaurene via *ent*-copalyl diphosphate (CPP). *ent*-Kaurene is oxidized to *ent-*kaurenoic acid and then to GA_12_ via several intermediates by two multifunctional cytochrome P450 (CYP450) monooxygenases, *ent*-kaurene oxidase (KO) and *ent*-kaurenoic acid oxidase (KAO), respectively. A third CYP450 converts GA_12_ to GA_53_ by hydroxylation on C-13. These C_20_ intermediates are converted in parallel pathways by soluble dioxygenases to the C_19_-GAs GA_9_ and GA_20_, respectively, and then to the bioactive phytohormones GA_4_ and GA_1_. The loss of a C atom (C-20) in the formation of C_19_-GAs occurs from an aldehyde intermediate (Kamiya and Graebe 1983). In contrast to plants, in which 13-hydroxylation (in GA_53_ formation) occurs early in the pathway and 3β-hydroxylation is the final step, in *F. fujikuroi*, 3β-hydroxylation occurs earlier, while 13-hydroxylation is the last step in the formation of GA_3_ (Bearder et al. 1975; Evans and Hanson 1975).

Further metabolism of inactive products is critical to the regulation of GA concentration. The most important inactivation process is 2-oxidation, which can occur on the bioactive GAs and their immediate C_19_ precursors, as well as on earlier C_20_ intermediates. Oxidation of C_19_-GAs to 2β-hydroxy products is especially strong in late-developing legume seeds, including pea seeds, in which further oxidation on C-2 to GA-catabolites was noted (Sponsel 1983).

### GA-biosynthesis mutants

GA-responsive dwarf mutants with lesions in the GA-biosynthetic pathway (Fig. 1) proved extremely useful in understanding GA physiology, identifying the underlying enzymes, and isolating the corresponding genes. Collections of single gene mutants of maize and peas were assembled by Phinney at University of California, Los Angeles (UCLA) and Murfet at Hobart, Tasmania, respectively, and the application of precursors revealed the positions of the lesions in the pathway. Such experiments established that GA_1_, but not its biosynthetic precursors, had biological activity in maize (Phinney and Spray 1982) and that *DWARF-1* in maize and *LE* in pea encode 3β-hydroxylases that convert GA_20_ to GA_1_ (Ingram et al. 1984; Spray et al. 1984). The *le* mutation corresponded to one of the traits, the difference in stem length, used in Mendel's classical experiments. Once the gene was identified, the mutation was shown to cause an amino acid substitution close to the Fe binding site that reduced enzyme activity (Lester et al. 1997; Martin et al. 1997). The *dwarf-5* mutation alters the activity of *ent*-kaurene synthase (KS) to produce mainly *ent-iso*kaurene, as shown in a cell-free system from maize seedlings (Hedden and Phinney 1979). The *slender* (*sln*) pea mutant illustrates the importance of GA inactivation in regulating GA concentration. The mutation is associated with excessive seedling growth, which decreases later in development (Reid et al. 1992; Ross et al. 1995). The gene, which encodes a GA 2-oxidase, is highly expressed in developing seeds, particularly in the testae (Lester et al. 1999; Martin et al. 1999). Mature pea seeds contain high levels of inactivation products oxidized on C-2 (Sponsel 1983), but *sln* seeds accumulate the precursor GA_20_, which upon germination is converted to GA_1_, causing the overgrowth symptoms (Reid et al. 1992).

In addition, Koornneef and van der Veen (1980) produced 56 independent GA-sensitive Arabidopsis (*Arabidopsis thaliana*) mutants, representing 5 loci, through irradiation- or EMS-induced mutagenesis. Mutations at three loci, named *ga1*, *ga2*, and *ga3*, prevented germination and caused severe dwarfism. By contrast, the other 2 mutations, *ga4* and *ga5*, allowed germination without GA treatment and produced only mild dwarfism. It was later shown that *GA1*, *GA2*, and *GA3*, which encode CPP synthase (CPS), KS, and KO, respectively, are single-copy genes, while the mild phenotypes of *ga4* and *ga5*, with impaired GA 3-oxidation and GA 20-oxidation, respectively, are due to gene redundancy (see below).

### Characterizing the enzymes of GA metabolism

Work in Charles West's laboratory at UCLA with cell-free preparations from *Marah macrocarpus* endosperm and *F. fujikuroi* mycelia led to the characterization of the enzymes involved in the conversion of GGPP to *ent*-kaurene. Using the *F. fujikuroi* system, Shechter and West (1969) showed that the conversion occurred in two steps, with CPP as an intermediate. The 2 activities, named activity A for conversion of GGPP to CPP and activity B for conversion of CPP to *ent*-kaurene, were found (after purification) to be present in a single polypeptide (Fall and West 1971). However, they are separate enzymes in *M. macrocarpus* but probably function in association (Duncan and West 1981). Activity A was renamed as the type II terpene cyclase CPS, while activity B, a type I cyclase, was renamed KS (MacMillan 1997). Early indications that these activities were present in plastids (Simcox et al. 1975) were later confirmed (Aach et al. 1995) and further substantiated by the presence of plastid-targeting leader sequences in CPS and KS (Sun and Kamiya 1994; Yamaguchi et al. 1996) and by the plastid localization of enzyme fusions with green fluorescent protein (GFP) (Helliwell et al. 2001b).

The enzyme activities responsible for the middle section of GA biosynthesis, from *ent*-kaurene to GA_12_ and GA_53_ in plants and to GA_14_ (3β-hydroxy GA_12_) in *F. fujikuroi*, were present in microsomes from cell-free systems from *M. macrocarpus*, pumpkin (*Cucurbita maxima*) endosperm, developing pea cotyledons, and the fungal mycelia and required NADPH (West 1973; Hasson and West 1976; Ropers et al. 1978; Graebe et al. 1980). These enzymes have the properties of CYP450s, which was confirmed when cDNAs encoding the enzymes were isolated (see below). By contrast, the final reactions in the pathway are catalyzed by soluble oxidases requiring Fe^2+^ and are therefore different from the monooxygenases responsible for earlier steps. The pumpkin enzymes were shown to require a small molecule whose identification as 2-oxoglutarate established the enzymes catalyzing GA_12_-aldehyde 7-oxidation, GA 20-oxidation, 3β-hydroxylation, and 2β-hydroxylation as 2-oxoglutarate-dependent dioxygenases (2-ODDs) (Hedden and Graebe 1982). The soluble 7-oxidase has restricted distribution between plant families, with most plants employing only a monooxygenase for this reaction, while in pumpkin endosperm, this reaction is catalyzed by both monooxygenase and dioxygenase enzymes.

Although work with cell-free homogenates demonstrated the efficient conversion of MVA into GAs, the application of ^13^C-labeled substrates to Arabidopsis seedlings indicated that *ent*-kaurene and GA_12_ were synthesized mainly from the MEP pathway with a small contribution from the MVA pathway (Kasahara et al. 2002). The extent of cross-over between these pathways, which is dependent on the movement of isoprenoid intermediates into and out of the plastid, may vary among tissues and developmental stages.

### Identification of genes encoding GA-metabolic enzymes

The isolation of transcripts and genes encoding the GA-biosynthetic enzymes were major developments that advanced our understanding of the regulation of GA metabolism. Sun et al. (1992) took advantage of the large deletion in the *ga1-3* Arabidopsis mutant to isolate the *GA1* gene by genomic subtraction. Expression of its cDNA in *Escherichia coli* demonstrated that it encodes CPS (Sun and Kamiya 1994). The maize gene *ANTHER EAR1* (*AN1*), which also encodes CPS, was isolated by tagging with the Mutator transposon shortly thereafter (Bensen et al. 1995). After purifying a GA 20-oxidase from pumpkin endosperm and partial amino acid sequencing (Lange 1994), the use of antibodies raised against synthetic peptides led to the isolation of its cDNA from an expression library (Lange et al. 1994). Expression in *E. coli* confirmed its enzymatic activity as oxidizing C-20 mainly to the carboxylic acid. Based on the nucleotide sequence of the pumpkin transcript, three *GA20ox* cDNAs were isolated from Arabidopsis and shown (by expression in *E. coli*) to encode enzymes that convert GA_12_ to the C_19_-GA, GA_9_ (Phillips et al. 1995). The tissue-specific expression patterns of the genes differed, but these genes showed partial redundancy, explaining the mild phenotype of the *ga5* mutant (Rieu et al. 2008b). Their expression was down-regulated by GA, confirming feedback regulation, which had been proposed earlier (Hedden and Croker 1992). A similar approach was used to clone one of these genes, *AtGA20ox1*, which corresponds to *GA5* (Xu et al. 1995). Arabidopsis contains five *GA20ox* genes, but only three of these, *AtGA20ox1,2,* and *3*, play major roles in plant development (Plackett et al. 2012).

The cloning of other GA-biosynthetic genes quickly followed. The Arabidopsis *GA4* gene was cloned by T-DNA tagging (Chiang et al. 1995) and confirmed (by heterologous expression) to encode a GA3ox (Williams et al. 1998). Of the four Arabidopsis *GA3ox* genes, two genes, *AtGA3ox1* and *AtGA3ox2*, regulate vegetative growth (Mitchum et al. 2006). Like *AtGA20ox1,2,* and *3*, *AtGA3ox1* is down-regulated by GA signaling as part of GA homeostasis (Cowling et al. 1998). *KS* was cloned from pumpkin following the purification of KS protein from cotyledons (Yamaguchi et al. 1996). This led to the isolation of *KS* cDNA from Arabidopsis and the finding (by mutant complementation) that it corresponded to *GA2* (Yamaguchi et al. 1998). Helliwell et al. (1998) demonstrated that *GA3* encodes KO based on the accumulation of *ent*-kaurene in the *ga3-1* mutant and its inability to respond to *ent*-kaurene application. The authors used map-based cloning and random sequencing to isolate *GA3*, which encodes a CYP450, and confirmed its identity by mutant complementation and by demonstrating KO activity after expression in yeast. *KAO* was cloned from barley (*Hordeum vulgare*) in which it corresponds to *GA-RESPONSIVE DWARF5* (*GRD5*), whose mutants accumulate *ent*-kaurenoic acid (Helliwell et al. 2001a). *GRD5* and 2 Arabidopsis homologs encode CYP88A family members, which, after expression in yeast, were shown to catalyze the 3-step conversion of *ent*-kaurenoic acid to GA_12_ via *ent*-7α-hydroxykaurenoic acid and GA_12_-aldehyde (Helliwell et al. 2001a). The equivalent enzyme in *F. fujikuroi* (CYP68A) also has 3β-hydroxylase activity and produces GA_14_ (Rojas et al. 2001). The two *AtKAO* genes share fully redundant functions, with the double mutant being severely dwarfed (Regnault et al. 2014). Thus, two enzymes, KO and KAO, are required to convert *ent*-kaurene to GA_12_ in plants, with a third CYP enzyme catalyzes the 13-hydroxylation of GA_12_ to GA_53_ (see below). Fusions of AtKO and AtKAO with GFP localized to the outer chloroplast envelope and endoplasmic reticulum (ER), respectively, provide a mechanism for the transit of *ent*-kaurene from plastids to the ER (Helliwell et al. 2001b).

The first cloning of a *GA2ox* cDNA took advantage of the very high GA2ox activity in late-developing *P. coccineus* seeds by functional screening of a cDNA expression library for release of ^3^H from [2β, 3β-^3^H_2_]GA_9_ (Thomas et al. 1999). The functions of the *P. coccineus* cDNA and three homologous Arabidopsis cDNAs identified in genomic databases were determined by expression in *E. coli.* The enzymes converted C_19_-GAs to their 2β-hydroxy analogs and, depending on the substrate and paralog, catalyzed further oxidation to GA-catabolites. GA promoted the expression of two of the Arabidopsis *GA2ox* genes, whereas it had the opposite effect on *GA20ox* and *GA3ox* gene expression. Like *GA20ox* and *GA3ox*, the *C_19_-GA2oxs* form a gene family with five functional members in Arabidopsis (Rieu et al. 2008a). Soon after, similar approaches were used to clone *GA2ox* cDNAs from developing pea seeds, one of which, *PsGA2ox1*, corresponds to *SLN* (Lester et al. 1999; Martin et al. 1999). A second clade of *GA2ox* genes with 2 members was identified from Arabidopsis by activation tagging, encoding enzymes that act on C_20_-GAs (Schomburg et al. 2003). Two additional members of this clade were identified in Arabidopsis recently (Lange et al. 2020). Apart from the *GA7ox* genes with restricted distribution, angiosperms contain four families of 2-ODDs, GA20ox, GA3ox, C_19_-GA2ox, and C_20_-GA2ox, while this last clade is absent from gymnosperms (Yoshida et al. 2020). The *C_19_-GA2ox* gene family is the largest in most species, with some tissue-specific expression but considerable redundancy.

GA 13-hydroxylase genes were first identified in rice. The encoded monooxygenases CYP714B1 and CYP714B2 converted GA_12_ to GA_53_ following expression in yeast (Magome et al. 2013). Overexpression of these genes in Arabidopsis caused a slight reduction in height, suggesting that 13-hydroxylation is a mild inactivation reaction. Other members of the CYP714 family oxidize 13-deoxy GAs and/or *ent*-kaurenoic acid on C-13 or adjacent C atoms and are also inactivating. Mutation of the rice enzyme CYP714D1, known as ELONGATED UPPERMOST INTERNODE (EUI), which oxidizes the 16,17-double bond to the epoxide, is utilized in hybrid rice production to promote panicle emergence in male-sterile cultivars (Zhu et al. 2006). Arabidopsis, which produces low amounts of 13-hydroxy GAs, except in the seed, contains two *CYP714* genes, *CYP714A1* and *CYP714A2*. Overexpression of CYP714A1, which converts GA_12_ to 16α-carboxy-17-norGA_12_, causes extreme dwarfism, while CYP714A2 functions mainly as a 12α-hydroxylase, with only low 13-hydroxylase activity, and causes mild dwarfism when overexpressed (Nomura et al. 2013). Notably, AtCYP72A9 was found to 13-hydroxylate GA_12_, GA_9,_ and GA_4_ (following expression in yeast) and may be the main source of 13-hydroxy GAs in Arabidopsis seeds (He et al. 2019). Its overexpression resulted in dwarfism.

### Regulation of GA metabolism

**Figure 3.**
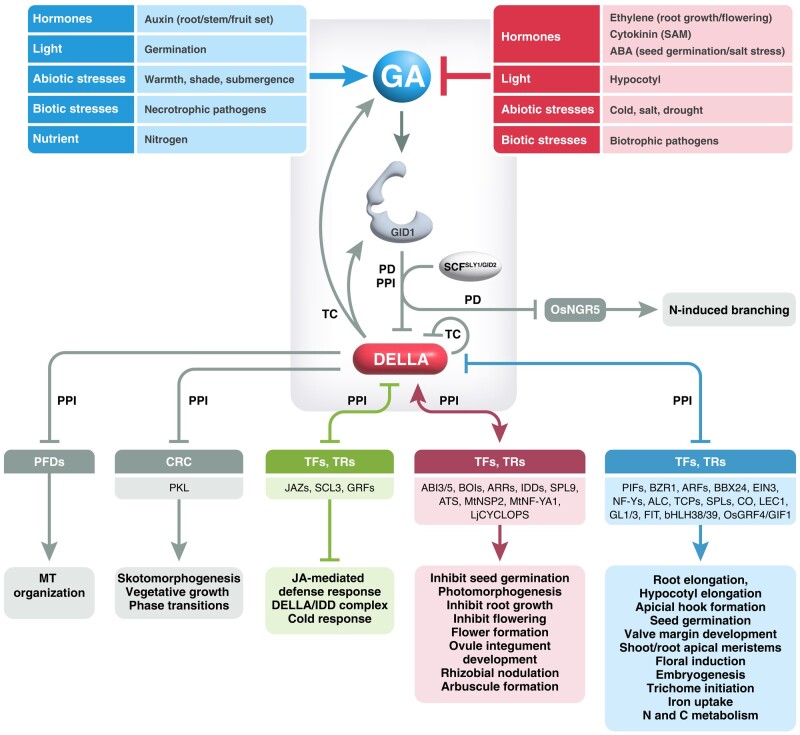
Interaction network between the GA-GID1-DELLA signaling module and various internal and external cues. Signals that increase bioactive GA levels are labeled in blue, while signals that decrease GA levels are shown in red. GA-GID1 triggers DELLA degradation via SCF^SLY1/GID2^-mediated polyubiquitination. GA-GID1 also induces OsNGR5 degradation in a SCF^GID2^-dependent manner to inhibit nitrogen-induced shoot branching in rice. DELLAs interact antagonistically or additively with a myriad of transcription factors (TFs), transcription regulators (TRs), CRC, and PREFOLDINs (PFDs) to modulate specific developmental processes. Most of the DELLA interactors are Arabidopsis proteins, except those that are labeled (Os, *Oryza sativa*; Mt, *Medicago truncatula*; Lj, *Lotus japonicus*). See Boxes 1 and 2 for details. PD, protein degradation; PPI, protein–protein interaction; TC, transcription; SAM, shoot apical meristem; MT, microtubule; GA, gibberellin; ABA, abscisic acid.

GA biosynthesis and inactivation are tightly regulated by developmental and environmental cues (Yamaguchi and Kamiya 2000; Sun 2008; Yamaguchi 2008; Hedden and Thomas 2012; Hedden 2020; Bouré and Arnaud 2023). Following the identification of genes encoding the metabolic enzymes, there have been numerous reports of their transcriptional regulation, particularly for the 2-ODDs, which limit the production of bioactive GAs. As discussed under GA perception and signaling, their expression is modified by other hormones as well as by numerous environmental factors, including stress (Fig. 3). While there is some evidence for post-transcriptional regulation (Lee and Zeevaart 2007), this process has been little studied for practical reasons. Reports of altered GA levels in GA-response mutants were early indications that GA-metabolism was regulated via GA signaling (reviewed in Hedden and Sponsel 2015). These observations highlighted 20-oxidation as a potential site of regulation by GA action, which was confirmed by experiment (Hedden and Croker 1992). Subsequently, as noted above, expression of some *GA20ox* and *GA3ox* genes was found to be repressed by GA, while *GA2ox* expression was upregulated (Thomas et al. 1999). Furthermore, down-regulation of *GA INSENSITIVE DWARF1* (*GID1*) receptor genes by GA extended homeostasis to GA signaling (Griffiths et al. 2006). The involvement of the DELLA GA-signaling component in this process is discussed below. A nontranscriptional homeostatic mechanism was revealed from the X-ray crystal structure of OsGA2ox3 (Takehara et al. 2020). In the presence of its substrate GA_4_, the enzyme forms a tetramer, thereby increasing its catalytic efficiency.

### GA biosynthesis in nonseed plants, fungi, and bacteria

In the evolution of land plants, DELLA-mediated signaling predates its regulation by GA-GID1 (Hernandez-Garcia et al. 2021). The evolution of GA biosynthesis was discussed in detail recently (Yoshida et al. 2020). The bryophyte *Physcomitrium patens* produces *ent*-kaurenoic acid derivatives but not GAs (Miyazaki et al. 2018), whereas the lycophyte *Selaginella moellendorffii* and the fern *Lycodium japonicum* produce GA_4_ using 2-ODDs for 20ox and 3ox activities and possess the GA-GID1-DELLA signaling system (Hernandez-Garcia et al. 2019; Yoshida et al. 2020). Notably, while *P. patens* and *L. japonicum* have bifunctional CPS/KS enzymes, *S. moellendorffi* has separate CPS and KS enzymes for *ent*-kaurene synthesis, as in seed plants. GA-inactivation by 2-oxidation is not present in nonseed plants and was acquired just before the establishment of gymnosperms (Yoshida et al. 2020). GA 2β-hydroxylation is also absent from GA-producing fungi and bacteria, whose genes for GA-biosynthesis are clustered in operons and were acquired independently of plants and each other (Hedden et al. 2001; Nett et al. 2017b). The members of both kingdoms employ CYP450s rather than 2-ODDs for 3- and 20-oxidation, while *F. fujikuroi* uses a 2-ODD to convert GA_4_ to GA_7_ in GA_3_ biosynthesis (Bhattacharya et al. 2012). Bacteria have separate CPS and KS enzymes, whereas fungi contain a bifunctional CPS/KS (Morrone et al. 2009). The capacity to produce bioactive GAs appears to be related to pathogenicity. Most symbiotic N-fixing bacteria produce GA_9_, allowing the plant to regulate GA_4_ production, while the phytopathogenic species *Xanthomonas oryzae* contains an extra gene encoding a 3β-hydroxylase (CYP115) and produces GA_4_ (Nagel and Peters 2017; Nett et al. 2017a).

## GA perception and signaling

### Dwarf and slender mutants with reduced or elevated GA responses

Genetic analyses of mutants displaying altered GA responses and molecular cloning of their corresponding genes have been instrumental in unveiling the long-sought GA receptor (GID1) and its immediate downstream repressors (DELLAs) and activators (F-box proteins GID2/SLEEPY1 [SLY1]). GA-unresponsive recessive mutants exhibit a dark-green, dwarf phenotype that mimics GA biosynthesis mutants, but their growth cannot be restored by GA treatment (Fig. 1). Plants with defects in genes encoding the GA receptor GID1 or the F-box protein GID2/SLY1 belong to this mutant class (McGinnis et al. 2003; Sasaki et al. 2003; Ueguchi-Tanaka et al. 2005). Gain-of-function DELLA mutants are dominant GA-unresponsive dwarves, e.g. *GA insensitive* (*gai*) in Arabidopsis (Koornneef et al. 1985) and *Reduced height* (*Rht*) varieties in wheat (Fig. 1), which were major contributors to the “Green Revolution” by increasing grain yield in the 1960s to 1970s (Börner et al. 1996; Peng et al. 1999). Conversely, “slender” mutants with elevated GA responses (e.g. *la cry* in pea and *slender* (*sln*) in barley) are recessive and display a tall and thin stem phenotype, which resembles wild-type plants that have been overdosed with GA (Brian 1957; Foster 1977).

Besides genetic analysis, cereal aleurone was also used extensively to study GA signaling (Lovegrove and Hooley 2000; Sun and Gubler 2004). During seed germination, aleurone cells produce hydrolytic enzymes in response to GA (diffused from the embryo) to degrade the starchy endosperm. Using this aleurone system, pharmacological assays have identified GA-induced genes and GAMYB, the transcription factor responsible for this transcriptional induction.

### GA-GID1 induces SCF^SLY1/GID2^-mediated DELLA degradation

With the development of Arabidopsis as a model system for plant research in the 1980s, the *gai-1* mutant and *repressor of ga1-3* (*rga*) mutants in Arabidopsis were isolated by screening for GA-unresponsive dwarves (Koornneef et al. 1985) or suppressors of the dwarf phenotype of *ga1-3* (Silverstone et al. 1997b), respectively. Intragenic suppressors of the semidominant *gai-1* mutant were generated by Ds transposon insertion mutagenesis (Peng and Harberd 1993), which guided the cloning of *GAI* (Peng et al. 1997). Suppressor mutants of *ga1-3* generated by fast-neutron mutagenesis contained large deletions in the *RGA* locus, which facilitated the cloning of *RGA* by genomic subtraction (Silverstone et al. 1998). The Arabidopsis genome contains five DELLA genes, *RGA*, *GAI*, *RGA-LIKE1* (*RGL1*), *RGL2*, and *RGL3*, which belong to a subfamily of GRAS (for GAI, RGA and SCARECROW) transcription regulators. The N-terminal DELLA domain is unique to the DELLA subfamily, whereas the C-terminal GRAS domain is shared among all GRAS family members (Pysh et al. 1999; Sun and Gubler 2004). Further characterization of orthologs in other species showed that DELLA genes are conserved in plants, including *Rht* in wheat (Peng et al. 1999), *SLENDER RICE1* (*SLR1*) in rice (Ikeda et al. 2001), *SLN1* in barley (Chandler et al. 2002), *D8* and *D9* in maize (Peng et al. 1999; Lawit et al. 2010), *PROCERA* in tomato (*Solanum lycopersicum*) (Jasinski et al. 2008), and *LA* and *CRY* in pea (Weston et al. 2008).

How are DELLA proteins regulated by the GA signal? The *la cry* slender mutant phenotype resembles the effects of GA overdose, leading to the idea that LA and CRY control the production of inhibitors (for GA-induced growth) and suggesting that GAs may function as “inhibitors of inhibitors” to promote growth (Brian 1957). Importantly, an examination of RGA protein levels by immunoblot analysis and confocal microscopy revealed that RGA protein levels in planta rapidly decreased in response to GA treatment (Silverstone et al. 2001). Furthermore, the *gai-1* Arabidopsis mutant encodes a mutant protein with a 17-amino acid deletion within the DELLA motif, suggesting that this mutation turns the GAI protein into a constitutive repressor of GA signaling (Peng et al. 1997). Indeed, deletion of the identical DELLA motif in RGA (rga-Δ17) abolished GA-induced degradation and conferred a GA-unresponsive dwarf phenotype, indicating that the DELLA motif is required for its proteolysis in response to GA (Dill et al. 2001). Notably, the semidwarf varieties of wheat and maize were found to be caused by deletion mutations in the DELLA domain of *Rht* and *D8*, respectively (Peng et al. 1999). Further studies of the recessive GA-unresponsive dwarf mutants Arabidopsis *sly1* and rice *gid2* led to the identification of F-box proteins and associated Skp, Cullin, F-box (SCF) ubiquitin E3 ligase complexes (SCF^SLY1/GID2^) that are responsible for polyubiquitination of DELLA and degradation by the 26S proteasome (Steber et al. 1998; McGinnis et al. 2003; Sasaki et al. 2003).

Although Arabidopsis research led to the breakthrough discoveries of the DELLA repressors and SLY1 (F-box) activators of GA signaling, the GA receptor remained elusive despite the efforts of multiple mutant screens. Eventually, aided by the completion of the rice genome sequence (Goff et al. 2002; Yu et al. 2002), the GA receptor was unveiled by positional cloning of *GA-insensitive dwarf1* (*gid1*) mutants in rice (Ueguchi-Tanaka et al. 2005). Notably, the smaller genome of Arabidopsis contains three *GID1* orthologs (*GID1A*, *GID1B*, and *GID1C*), whose functional redundancy explains why genetic screens failed to identify GA receptors in Arabidopsis (Griffiths et al. 2006; Nakajima et al. 2006). GID1 is localized to both the cytoplasm and nucleus and belongs to the hormone-sensitive lipase (HSL) family, although it lacks one of three key catalytic residues for lipase activity. Yeast two-hybrid (Y2H) and in vitro pull-down assays show that GA binding to GID1 promotes GID1-DELLA interactions (Ueguchi-Tanaka et al. 2005) and that the DELLA domain (including DELLA, LEXLE and VHYNP motifs) is essential for its interaction with GID1 (Griffiths et al. 2006; Willige et al. 2007). Molecular details of the GA-AtGID1A-DELLA domain (GAI) complex and GA-OsGID1 determined by X-ray crystallography revealed that bioactive GA is an allosteric inducer of its receptor GID1 (Murase et al. 2008; Shimada et al. 2008). The carboxy-terminal core domain of GID1 forms a GA-binding pocket, and the amino-terminal extension (N-Ex) domain acts as a lid. GA binding induces a conformational switch of its N-Ex to close the GA-binding pocket and creates hydrophobic surfaces for DELLA binding (Fig. 2).

How does GA-GID1-DELLA promote SLY1/GID2 recognition? Mutant and Y2H analyses indicated that SLY1/GID2 interacts with the GRAS domain of the DELLA protein (Dill et al. 2004; Hirano et al. 2010). Yeast 3-hybrid assays further demonstrated that GA-bound GID1 enhances the RGA–SLY1 interaction, suggesting that the GA/GID1-DELLA domain interaction triggers conformational changes in the GRAS domain for SLY1 recognition (Griffiths et al. 2006). Moreover, the GRAS domain of SLR1 was shown to interact with GID1 after the binding of the DELLA domain to further stabilize the GID1-SLR1 complex, which allows efficient recognition by the F-box protein GID2 (Hirano et al. 2010).

### Mechanism of DELLA action: transcriptional reprograming via protein–protein interactions with hundreds of transcription factors

DELLAs are nucleus-localized transcription regulators. Transcriptome studies on early GA- and DELLA-responsive genes showed that DELLAs can activate or repress transcription, depending on the target genes (Zentella et al. 2007; Hou et al. 2008). DELLAs do not contain any canonical DNA binding motifs and have not been shown to bind DNA directly. Importantly, chromatin immunoprecipitation (ChIP)-qPCR analysis demonstrated an association of RGA with its target chromatin (Zentella et al. 2007). ChIP-sequencing (seq) analysis identified genome-wide RGA binding sites: 421 associated genes in Arabidopsis seedlings and 2,327 associated genes in the inflorescence meristem (Marin-de la Rosa et al. 2015; Serrano-Mislata et al. 2017). Abundant evidence indicates that DELLAs regulate transcription via antagonistic or additive interactions with a myriad of transcription factors/regulators (Figs. 2 and 3 and Boxes 1 and 2) (Sun 2011; Daviere and Achard 2016; Van De Velde et al. 2017). PHYTOCHROME INTERACTING FACTORS (PIFs), PIF3, and PIF4 were the first reported DELLA-interacting transcription factors (TFs) (de Lucas et al. 2008; Feng et al. 2008). PIF3 and PIF4 are light-responsive bHLH TFs that promote hypocotyl elongation. Genetic analysis and ChIP-qPCR showed that the DELLA-PIF3/4 interaction sequesters PIFs from binding to the promoters of growth-related genes, revealing molecular crosstalk between light and GA signaling. Extensive studies in the last 15 years have identified 370 potential DELLA-interacting proteins in Arabidopsis by Y2H screens, and over 40 of them have been verified by co-IP and/or genetic analyses (Marin-de la Rosa et al. 2014; Van De Velde et al. 2017; Lantzouni et al. 2020).

**Box 1. DELLA Interactors: Transcription factors/regulators**

(1) DELLA-repressed transcription activators include several TFs that promote hypocotyl elongation: PIF3/4 (light signaling regulators), BRASSINAZOLE-RESISTANT1 (BZR1, a brassinosteroid signaling activator) (Bai et al. 2012; Gallego-Bartolome et al. 2012), AUXIN RESPONSE FACTORs (ARFs, auxin signaling activators) (Oh et al. 2014), and BBX24 (B-box zinc finger protein) (Crocco et al. 2015). DELLAs also inhibit the activities of ETHYLENE INSENSITIVE3 (EIN3, an ethylene signaling activator) in apical hook formation (An et al. 2012); NUCLEAR FACTOR Ys (NF-Ys) in seed germination and flowering (Hou et al. 2014); ALCATRAZ (ALC, bHLH) in fruit valve margin development (Arnaud et al. 2010); Type I TCPs (TEOSINTE BRANCHED 1 [TB1], CYCLOIDEA [CYC], and PROLIFERATING CELL FACTOR [PCF]) in cell division in shoot and root apical meristems (Daviere et al. 2014; Resentini et al. 2015); GLABRA1 (GL1, MYB23) and GL3 (bHLH) in trichome initiation (Qi et al. 2014); SQUAMOSA PROMOTER BINDING PROTEIN LIKEs (SPLs) and CONSTANS (CO) in floral induction (Yu et al. 2012; Hyun et al. 2016; Xu et al. 2016); LEAFY COTYLEDON1 (LEC1 = NF-YB9) in late embryogenesis (Hu et al. 2018); FIT (bHLH) and bHLH38/39 for iron uptake in the root (Wild et al. 2016); and the GROWTH REGULATING FACTOR4 (OsGRF4)/GRF-Interacting Factor1 (OsGIF1) complex in nitrogen and carbon metabolism and nitrogen uptake (Li et al. 2018).

(2) DELLA-activated transcription factors/regulators include ABSCISIC ACID INSENSITIVE 3 (ABI3) and ABI5 (a bZIP TF), which mediate ABA-inhibited seed germination (Lim et al. 2013); BOTRYTIS SUSCEPTIBLE1 INTERACTORs (BOIs, RING domain protein), which inhibit seed germination, the juvenile-to-adult transition, and floral induction (Park et al. 2013); type-B ARRs, which function in cytokinin-induced de-etiolation and root meristem cell division (Marin-de la Rosa et al. 2015); the INDETERMINATE DOMAIN (IDD) subfamily of C2H2 zinc finger TFs, which regulate root development, inhibit floral induction, and regulate GA homeostasis (Fukazawa et al. 2014; Yoshida et al. 2014); SPL9, which promotes flower formation; ABERRANT TESTA SHAPE (ATS = KANADI4, KAN4), which promotes ovule integument development (Gomez et al. 2016); NODULATION SIGNALING PATHWAY2 (MtNSP2, a GRAS protein) and MtNF-YA1 in *Medicago truncatula*, which promote rhizobial nodulation (Fonouni-Farde et al. 2016; Jin et al. 2016); and LjCYCLOPS in *Lotus japonicus*, which functions in arbuscule formation (Pimprikar et al. 2016).

**Box 2. DELLA Interactors: Transcription repressors and others**

(1) DELLA-repressed transcription repressors include the jasmonate (JA) signaling repressors JAZs, which promote JA-induced defense responses against herbivory and necrotrophs (Hou et al. 2010). Moreover, the JAZ–DELLA interaction inhibits DELLA-PIF3 to promote plant growth, revealing the role of JAZ/DELLA/PIF in balancing plant defense and growth (Yang et al. 2012). DELLAs also inhibit SCL3 (a GRAS protein) activity by interacting and competing with SCL3 for binding to IDDs (Zhang et al. 2011; Yoshida et al. 2014). SCL3 is an activator of GA signaling whose transcription is induced by DELLA but repressed by itself. In addition, DELLAs inhibit the activity of GRFs in promoting the expression of cold-induced genes (Lantzouni et al. 2020).

(2) DELLAs interact with CRC, including SWI3C (Sarnowska et al. 2013) and PICKLE (PKL) (Zhang et al. 2014). SWI3C is a core subunit of the Switch (SWI)/Sucrose Nonfermenting (SNF)-type CRC. Transcript analysis suggested that SWI3C promotes the expression of DELLA-induced genes (e.g. *GID1A* and *SCL3*), although the mechanism is unclear (Sarnowska et al. 2013). An antagonistic interaction between DELLA and PKL regulates GA-induced skotomorphogenesis, vegetative growth, and the phase transition (Zhang et al. 2014; Park et al. 2017).

(3) DELLAs sequester the cochaperones PREFOLDINs (PFDs) to the nucleus, which disrupts microtubule organization in the cytoplasm (Locascio et al. 2013).

DELLAs appear to function as transcription co-activators or corepressors, depending on which transcription factors/regulators they interact with. Three distinct modes of DELLA action have been reported: (i) DELLA represses transcription by blocking DNA binding and sequestering transcriptional activators (e.g. PIFs) from their target promoters; (ii) DELLA activates transcription by recruiting transcription factors (e.g. ABSCISIC ACID INSENSITIVE 3 [ABI3] and ABI5, ARABIDOPSIS RESPONSE REGULATORs (ARRs), and IDDs); and (iii) DELLA activates transcription by sequestering transcription repressors (e.g. JASMONATE ZIM DOMAINs [JAZs], SCARECROW-LIKE3 [SCL3]) from their target promoters (Figs. 2 and 3) (Daviere and Achard 2016; Thomas et al. 2016; Van De Velde et al. 2017). ChIP-seq analyses showed that RGA binding peaks are enriched near cis-elements for several DELLA-interacting TFs (Marin-de la Rosa et al. 2015; Serrano-Mislata et al. 2017), supporting the notion that DELLAs are recruited to target promoters by their interacting TFs.

### Multiple signaling pathways regulate DELLA-mediated plant responses to internal and environmental cues

DELLAs were initially identified as GA signaling repressors, and GA promotes rapid DELLA proteolysis mediated by GID1 and SCF^SLY1/GID2^ (Sun and Gubler 2004). Notably, DELLAs also play a key role in feedback regulation to help maintain GA homeostasis by inducing the transcription of genes encoding GA biosynthetic enzymes and GID1s (Fig. 3) (Zentella et al. 2007). However, extensive studies of DELLA interactors and DELLA-regulated processes in the last 15 years have unveiled a much broader function of DELLAs as master growth regulators that integrate the activities of many signaling pathways in response to developmental and external cues, including biotic and abiotic stress (Thomas et al. 2016; Van De Velde et al. 2017). Boxes 1 and 2, and Fig. 3 highlight the diverse processes regulated by DELLAs and their interactors. Importantly, DELLA activity can be regulated by several mechanisms: (i) altered DELLA stability (GA-GID1 dependent) by modulating GA metabolism; (ii) altered DELLA activity by interacting with TFs/TRs/chromatin-remodeling complexes (CRC); (iii) GA-GID1-independent polyubiquitination and degradation; (iv) other post-translational modifications (PTMs); and (v) transcriptional induction of *RGL3* by JA signaling.

(1) Altered DELLA stability (GA-GID1 dependent) by modulating GA metabolism

Factors that decrease bioactive GA levels to increase DELLA accumulation include other phytohormones (ABA, cytokinin, and ethylene), external cues (e.g. light, abiotic stresses [cold, salt, and drought], and biotic stress [biotrophic pathogens]) (Fig. 3). Conversely, factors that increase bioactive GA levels to decrease DELLA accumulation include auxin, and external cues (e.g. nitrogen, light, abiotic stresses [warmth, shade, submergence], and biotic stress [necrotrophic pathogens]). Most of these findings have been discussed in previous reviews (Sun 2011; Colebrook et al. 2014), except for two recent studies showing that drought inhibits GA biosynthesis in the leaf base of wheat seedlings (Ptošková et al. 2022) and that nitrate (a major nitrogen sources) induces root growth by increasing GA biosynthesis, resulting in reduced DELLA protein accumulation in Arabidopsis and wheat (Camut et al. 2021). Recent studies on the semidwarf Green Revolution varieties (GRVs) of wheat (*Rht* alleles encoding dominant DELLAs) and rice (*semi-dwarf1* [*sd1*], defective in GA20ox2) demonstrated that nitrogen use efficiency (NUE) is regulated by the GA signaling pathway (Wang et al. 2021; Liu et al. 2022). The GRVs dramatically increase crop yields, although they exhibit low NUE because SLR1 inhibits nitrogen and carbon metabolism, nitrogen uptake, and assimilation by disrupting the interaction between the key TF OsGRF4 and its coactivator OsGIF1 (Li et al. 2018) (Fig. 3). Notably, elevated DELLA activities in GRVs promote nitrogen-induced tillering (shoot branching). It turns out that GID1 and DELLA can interact with NITROGEN MEDIATED TILLER GROWTH RESPONSE 5 (NGR5), a transcription factor that represses the expression of genes that inhibit tillering (Wu et al. 2020). GA-GID1 promotes NGR5 degradation by the SCF^GID2^-mediated ubiquitin-proteasome pathway (Fig. 3), while DELLA competes with GID1-NGR5 interaction to stabilize NGR5.

(2) Altered DELLA activity by antagonistic or additive interactions with TFs/TRs

(Boxes 1 and 2, and Fig. 3).

(3) GA/GID1-independent DELLA degradation

**Figure 4.**
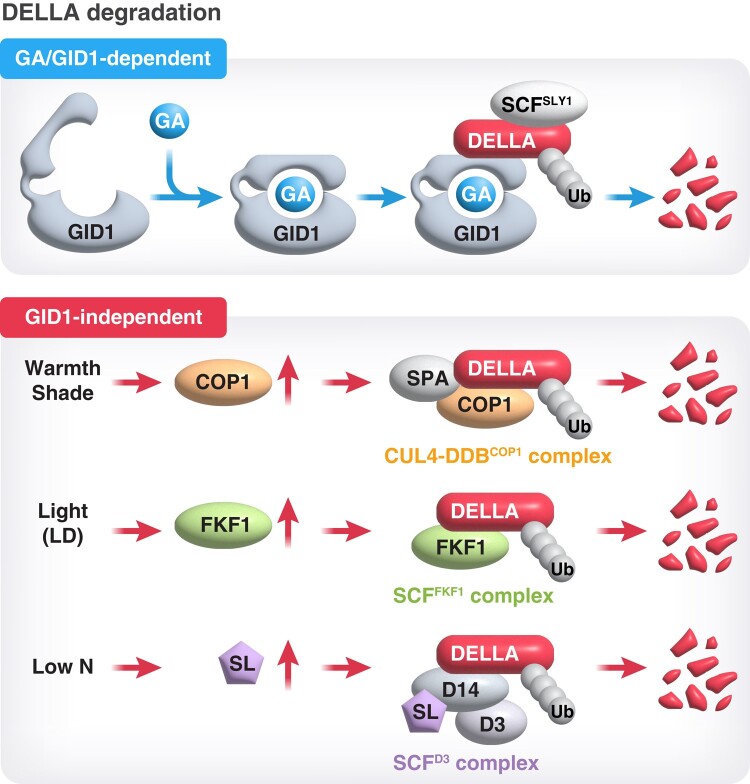
GA/GID1-dependent versus GA/GID1-independent DELLA degradation. GA/GID1-dependent proteolysis of DELLA is mediated by SCF^SLY1/GID2^. DELLA can be destabilized by signals including warmth, shade, LD light, and low nitrogen conditions. COP1 and its associated CUL4-DDB^COP1^ ubiquitin E3 ligase complex mediate warmth- and shade-induced DELLA degradation in Arabidopsis. The SCF^FKF1^ ubiquitin E3 ligase complex mediates LD-induced DELLA proteolysis in Arabidopsis. Low nitrogen conditions induce the biosynthesis of SL in rice, which binds to its receptor D14 and promotes the D14-DELLA interaction and DELLA degradation mediated by SCF^D3^. GA, gibberellin; Ub, ubiquitin.

Besides GA/GID1/SCF^SLY1/GID2^-dependent proteolysis, DELLA protein stability can be regulated by 3 other pathways (Fig. 4).

CONSTITUTIVE PHOTOMORPHOGENIC1 (COP1) mediates DELLA degradation in response to warm temperatures and shade:

Shade- or warm temperature-induced hypocotyl elongation correlates with elevated bioactive GA levels and a reduction in DELLA protein levels (Djakovic-Petrovic et al. 2007; Stavang et al. 2009; Arana et al. 2011). Surprisingly, the abundance of the GA-resistant rga-Δ17 protein is also reduced by these environmental cues, suggesting the presence of a GA/GID1-independent mechanism for DELLA degradation (Blanco-Tourinan et al. 2020a). Biochemical and genetic analyses showed that the E3 ubiquitin ligase COP1 and its interacting protein SUPPRESSOR OF phyA-105 proteins (SPAs) play a direct role in the rapid proteolysis of DELLA prior to changes in GA content in the shade or under warm conditions. As the liverwort (*Marchantia polymorpha*) genome contains putative orthologs of *COP1* and *DELLA* genes, COP1-mediated DELLA degradation may serve to regulate DELLA activity prior to the GA/GID1-mediated mechanism, which appears later in lycophytes.

FLAVIN-BINDING KELCH REPEAT F-BOX 1 (FKF1) mediates DELLA degradation to promote flowering in LD conditions:

FKF1 promotes flowering under long-day (LD) conditions. The *fkf1* mutant is late flowering and has elevated RGA protein levels even in the GA-deficient background (Yan et al. 2020). co-IP assays in *Nicotiana benthamiana* and in vitro assays showed that FKF1 directly binds to DELLAs and promotes their ubiquitination and degradation. It was proposed that FKF1 regulates the cyclical degradation of DELLA in LDs, but this remains to be verified.

Strigolactone (SL)-D14 mediates DELLA degradation in response to low nitrogen conditions:

As described above, GA reduces NUE by promoting NGR5 degradation. Conversely, SL increases NUE. D53 is a repressor of SL signaling that inhibits the expression of GRF4-induced genes for N metabolism. Low nitrogen conditions induce the biosynthesis of SL, which binds to its receptor D14 and promotes the D14-D53 interaction and subsequent SCF^D3^-mediated ubiquitination and degradation of D53. Notably, SL also promotes the interaction of D14 with SLR1 (rice DELLA) (Nakamura et al. 2013), which leads to SLR1 degradation mediated by SCF^D3^ (Sun et al. 2023). D53 competes with SLR1 for binding to D14, adding another layer of regulation for DELLA degradation.

(4) Regulation of DELLA Function by PTMs:

In addition to polyubiquitination, which promotes DELLA proteolysis, DELLA activity is also modulated by other PTMs, including Small Ubiquitin-like Modifier (SUMO)-conjugation (SUMOylation), phosphorylation, and *O*-glycosylation (*O*-linked *N*-acetylglucosamine [*O*-GlcNAc] and *O*-fucose modifications) (Blanco-Tourinan et al. 2020b; Sun 2021). Under salt-stress conditions, DELLA SUMOylation is induced due to increased degradation of the SUMO proteases OVERLY TOLERANT TO SALT 1 and 2 (OTS1 OTS2) (Conti et al. 2014). SUMOylated DELLA binds to and sequesters GID1 independently of GA, thereby promoting the accumulation of non-SUMO-DELLA and restricting plant growth. Under nonstress conditions, OTS-mediated de-SUMOylation of DELLA promotes the growth of stamen filaments (Campanaro et al. 2016). The role of phosphorylation in DELLA function is not well understood. An early study reported that GA-induced SLR1 degradation in rice occurs independently of phosphorylation (Itoh et al. 2005). However, another study suggested that the phosphorylation of SLR1 by the casein kinase I EARLIER FLOWERING1 (EL1) increases its stability (Dai and Xue 2010). The *el1* mutant displays elevated GA response and early flowering phenotypes, and the SLR1-YFP (yellow fluorescent protein) protein in *35S:SLR1-YFP el1* transgenic rice was degraded more rapidly after GA treatment than in the wild-type background. These findings suggest that EL1 may inhibit GA signaling by enhancing DELLA stability, although SLR1 phosphorylation by EL1 was only shown in vitro.

The discovery of the role of *O*-glycosylation in regulating DELLA activity came from the characterization of the Arabidopsis *spindly* (*spy*) mutants, which partially rescue the GA-deficient dwarf phenotype caused by a GA biosynthesis inhibitor (paclobutrazol) or a mutation (*ga1*), indicating that SPY is a repressor of GA signaling (Jacobsen and Olszewski 1993; Jacobsen et al. 1996; Silverstone et al. 1997b, 2007). Both SPY and its paralog SECRET AGENT (SEC) in Arabidopsis were predicted to be *O*-GlcNAc transferases (OGTs) based on sequence analysis (Olszewski et al. 2010). Both SPY and SEC contain a tetratricopeptide-repeat (TPR) domain and a putative OGT catalytic domain. Surprisingly, electron transfer dissociation (ETD)-MS/MS and in vitro enzyme assays showed that SPY *O*-fucosylates DELLAs and that SEC *O*-GlcNAcylates DELLAs (Zentella et al. 2016, 2017). Genetic analysis and pulldown assays further showed that *O*-fucosylation of DELLA by SPY enhances DELLA binding to TFs (e.g. BZR1 and PIFs), while *O*-GlcNAcylation of DELLA by SEC reduces DELLA activity. As OGT serves as a nutrient sensor in metazoans (Hart 2019), it was proposed that *O*-Fuc and *O*-GlcNAc modifications might modulate DELLA activity and plant growth in response to nutrient availability.

(5) Transcriptional induction of *RGL3* by JA signaling

*RGL3* transcription is rapidly induced by MYC2, which is a JA signaling activator (Wild et al. 2012). As RGL3 binds to and sequesters the JA signaling repressors JAZs, elevated expression of *RGL3* in response to the JA signal enhances MYC2 activity to promote JA-mediated resistance to necrotrophic pathogens.

### DELLA-independent GA responses

Although DELLAs control almost all GA-regulated processes, a few DELLA-independent GA responses have been reported. SPATULA (SPT), a bHLH TF that is unrelated to DELLAs, also inhibits GA-induced cotyledon expansion and fruit growth (Josse et al. 2011; Fuentes et al. 2012). The SPT-repressed cotyledon expansion is independent of light conditions, which is in contrast to DELLA, whose stability is reduced by red light-induced GA biosynthesis. Notably, DELLAs negatively regulate *SPT* transcript accumulation, which provides a balance between the two classes of repressors (Josse et al. 2011). Another DELLA-independent GA response is GA-induced increases in cytosolic Ca^2+^ levels, although it remains to be determined whether this GA response is mediated by GID1 in the cytoplasm (Okada et al. 2017).

## GA transport mechanism

### Long-distance GA transport

Nearly half a century ago, initial findings were reported on the movement of GA in plants (Zweig et al. 1961; Chin and Lockhart 1965; Hoad and Bowen 1968). These studies confirmed the presence of GA in the phloem sap and its ability to travel through this medium. Since then, ongoing efforts have been made to understand and measure the movement of GA in plants (Hedden and Sponsel 2015; Binenbaum et al. 2018). GA moves within the plant in both upward (acropetal) and downward (basipetal) directions (Proebsting et al. 1992; Bjorklund et al. 2007; Regnault et al. 2015; Lacombe and Achard 2016). The movement of GA is crucial for various developmental processes in plants (Anfang and Shani 2021; Zhang et al. 2023).

**Figure 5.**
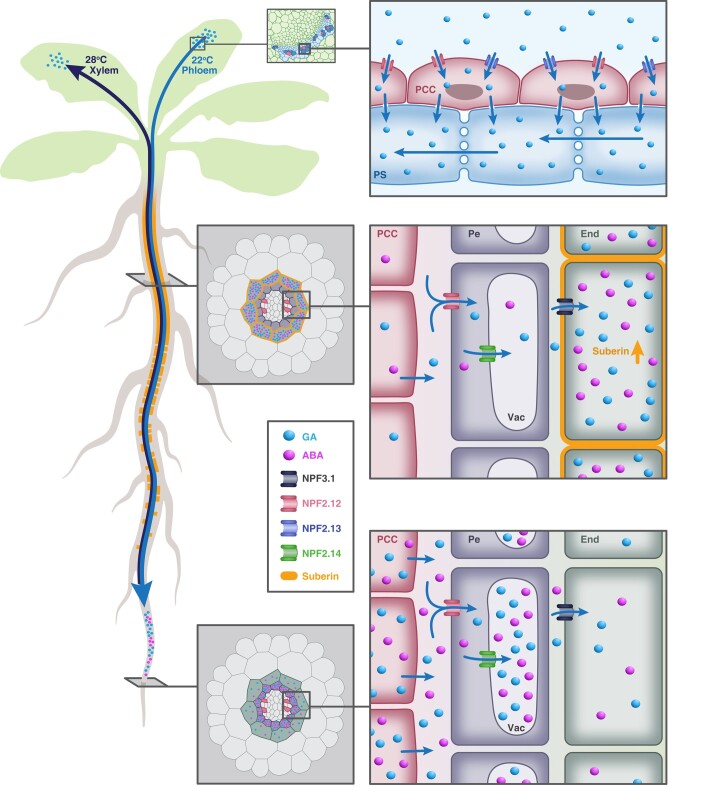
Model of the role of NPF2s activity in regulating shoot-to-root translocation of GA_12_ to promote endodermal suberin deposition. NPF2.12 and NPF2.13 are found in the phloem of the shoot and play vital roles in transporting GA_12_ over long distances from shoot to root. NPF2.12 is active in the membranes of root pericycle cells and facilitates the movement of ABA and GA from the vasculature to the pericycle region. Once inside the pericycle's cytoplasm, NPF2.14 transports these hormones into the vacuole, where they are possibly stored as a reserve for future use. As the root elongates over time and these cells mature, the stored hormones are released from the pericycle vacuole and taken up by the endodermis through the action of NPF3.1. This uptake triggers suberization, a process that forms a protective barrier in the endodermis. In addition, GA_12_ derived in the root, plays a role in regulating the shoot growth in response to ambient temperatures (28°C) in Arabidopsis. PCC, phloem companion cell; PS, phloem sieve; Pe, pericycle; End, endodermis; Vac, vacuole; GA, gibberellin; ABA, abscisic acid.

Several studies have attempted to identify the mobile form of GA by perturbing GA biosynthesis at different stages of the pathway. In peas, grafting mutant plants deficient in GA biosynthetic enzymes onto wild-type plants led to an increase in GA content in the shoots compared to non-grafted mutant plants. GA analysis revealed that GA_20_ was the major mobile form in pea plants (Proebsting et al. 1992). Similarly, in Arabidopsis, grafting experiments with mutant plants at different stages of GA biosynthesis identified GA_12_ as the major form transported over long distances through the vasculature (Regnault et al. 2015) (Fig. 5). GA_12_ moves through the xylem from roots to shoots and through the phloem from shoots to roots to regulate plant growth (Regnault et al. 2015). The transport activity is most evident in plants that fail to synthesize GA locally. It was furthermore demonstrated that GA_12_ derived in the roots plays a role in regulating the growth of shoots in response to temperature changes in Arabidopsis (Camut et al. 2019). Most recently, it was reported that two GA and ABA transporters (NPF2.12 and NPF2.13) are required for shoot-to-root GA_12_ translocation to regulate endodermal root suberization (summarized below) (Binenbaum et al. 2023).

### GA movement and localization

The synthesis of active GA is a complex and multistep process involving various intermediate compounds (Fig. 2). This complexity makes it challenging to identify the specific tissues or organs where GAs are produced and localized. Analysis of GA biosynthesis reporter lines indicated that while some tissues show colocalization of GA biosynthesis genes and GA perception genes, there are cases where these two groups of genes do not overlap. For example, GA biosynthesis genes are not expressed in the aleurone cells of the rice endosperm, but GA signaling genes are (Kaneko et al. 2003). This spatial separation suggests the need for GA movement within the plant. In addition, the expression levels of genes involved in the GA biosynthesis pathway itself do not always align. For instance, the expression of the late-stage GA biosynthesis genes *AtGA3ox1* and *AtGA3ox2* in germinating embryos differs spatially from that of the early GA biosynthesis gene *AtCPS* (Silverstone et al. 1997a; Yamaguchi et al. 2001). Similar patterns are found in different root cell types (Barker et al. 2021). Such differences indicate that the location and movement of GA precursors could play a vital role in regulating GA responses. An interesting example of GA precursor translocation was found in the fern *L. japonicum*, where GA movement is involved in the sex-determining mechanism. The study proposed a model in which different stages of prothalli in a colony express different GA-biosynthetic genes, producing specific forms of GA that regulate the formation of reproductive structures (Tanaka et al. 2014). In the future, there is a need to measure GA contents (including bioactive GAs and precursors) in different tissues and cell types at the single-cell level to shed light on the GA map with respect to its dynamic movement.

Long and short-distance movement of different GA forms are crucial for plant development (Rizza and Jones 2019; Wexler et al. 2019; Anfang and Shani 2021). For example, one of the functions of GA is to induce xylem differentiation in the hypocotyl following the floral transition in Arabidopsis. *GA3ox1* mRNA levels increase in the shoot but not the hypocotyl after flowering, suggesting GA movement. Mutant plants lacking GA biosynthesis, such as the *ga1-3* mutant, exhibit reduced hypocotyl xylem expansion after the flowering stage (Ragni et al. 2011). However, xylem expansion was restored when the mutant plants were grafted onto wild-type rootstocks. This suggests that GA acts as a mobile signal derived from the shoot that triggers xylem expansion in the hypocotyl (Ragni et al. 2011). Similar effects were observed in tobacco (*Nicotiana tabacum*) plants when defoliation occurred, resulting in reduced GA content and growth abnormalities in the stem (Dayan et al. 2012).

Additional reports have described the dependency of certain organs on external sources of GA. For example, GA movement from the embryo scutellum to the aleurone in cereal grains plays a pivotal role in regulating seed germination (Paleg 1960). Upon imbibition, during germination, the scutellum synthesizes GAs, which move to the aleurone layer (Hayashi 1940). In the aleurone, GA triggers the activation of hydrolytic enzymes that break down the stored starch and proteins in the endosperm into simpler forms, providing essential nutrients for the developing embryo (Lovegrove and Hooley 2000; Sun and Gubler 2004). This coordinated GA-mediated movement and response from scutellum to aleurone are fundamental for successful germination and early seedling growth in cereal grains.

In addition, petals rely on anthers as a source of GA for their growth and development (Hu et al. 2008). Studies in Arabidopsis, petunia (*Petunia hybrida*), tobacco, and rice demonstrated that GA produced in the anthers is crucial for petal development (Weiss and Halevy 1989; Itoh et al. 1999, 2001; Hu et al. 2008). These pieces of evidence support the idea that GA acts as a mobile plant hormone and that its movement is necessary for various processes involved in plant growth and development.

In cucumber flowers, specific forms of GA were found to be localized to different floral parts, suggesting their involvement in localized growth regulation. Experiments using deuterated GA provided quantitative support for the production and movement of GA from ovaries to sepals and petals, where it is converted to a bioactive form to regulate organ growth (Lange and Lange 2016). A study characterizing GA biosynthesis sites in roots at the cellular level, coupled with cell-type-specific GA synthesis rescue experiments, indicated GA movement between root cell-files (Barker et al. 2021). Analysis of photocaged bioactive GA_4_ released endogenously in Arabidopsis roots allowed the kinetic parameters of its flow to be measured, such as decay length and velocity (Wexler et al. 2019). More comprehensive studies are needed to explore additional developmental stages where GA movement plays a fundamental role by correlating the expression patterns of GA biosynthetic genes with direct measurements of GA levels.

### GA transporters

The first evidence of bioactive and regulated GA transport came with the identification of GA transporters from the NPF (Chiba et al. 2015; Saito et al. 2015; David et al. 2016; Tal et al. 2016; Anfang and Shani 2021; Kanstrup and Nour-Eldin 2022). Several NPF transporter proteins, including NPF2.3, NPF2.4, NPF2.5, NPF2.7, NPF2.10, and NPF3.1 have been identified as potential GA transporters using yeast-modified 2-hybrid systems and further confirmed in *Xenopus* oocytes (Chiba et al. 2015; Saito et al. 2015; Wulff et al. 2019). However, the physiological importance of most of these transporters in plants remains to be fully understood. The main challenge in characterizing GA transporters and their physiological function is the lack of apparent GA-mediated phenotypes in the respective mutants. Genetic redundancy plays a substantial role in this shortcoming, as most, if not all, transporter proteins belong to large and robust gene families. Thus, the knockout of one putative GA transporter is compensated for by another family member (Zhang et al. 2023).

Recent research has revealed that a subset of NPF proteins is required for the mechanisms behind long-distance GA transport from shoot to root and its developmental importance (Binenbaum et al. 2023). GA transport plays a critical role in suberin formation in the root. NPF2.12 and NPF2.13 (2 recently identified GA and ABA importers), along with NPF2.14 (a tonoplast importer), coordinate the regulation of suberin formation (Binenbaum et al. 2023). NPF2.12 and NPF2.13 are membrane-localized proteins expressed in leaf phloem companion cells that facilitate the transport of GA_12_ from shoot to root (Fig. 5). Once reaching the root, GA_12_ is converted to GA_4_ by the enzymes GA20ox and GA3ox. It is speculated that the bioactive GA and ABA exit the phloem at the phloem unloading zone (Robe and Barberon 2023) located around the root elongation zone (Binenbaum et al. 2023). NPF2.12 is then able to import GA_4_ and ABA into the pericycle, and subsequently, the pericycle-specific transporter NPF2.14 transports these phytohormones into the vacuole (Fig. 5) GA and ABA accumulate in the vacuole within the phloem unloading zone located around the root elongation zone, where they are stored during root maturation and differentiation. Only later in development are these plant hormones released from the vacuole by an unknown mechanism and are able to be taken into the endodermis by NPF3 to promote suberization. NPF3 is localized to the plasma membrane and imports GAs in a pH-dependent manner (David et al. 2016; Tal et al. 2016) (Fig. 5). These findings indicate that GA and ABA can work in a nonantagonistic manner to regulate plant development. This mechanism highlights the importance of long-distance shoot-to-root movement of GA_12_ and the accumulation of bioactive GA_4_ and ABA in the endodermis for regulating endodermal suberization (Binenbaum et al. 2023; Zhang et al. 2023).

In Arabidopsis, the Sugars Will Eventually Be Exported Transporters (SWEET) family members SWEET13 and SWEET14 have been identified as GA transporters (Kanno et al. 2016; Morii et al. 2020). These transporters import GAs, as demonstrated in yeast and oocyte transport assays (Kanno et al. 2016). SWEET13 and SWEET14 function redundantly to regulate anther development, and the application of exogenous GAs to the *sweet13 sweet14* double mutant rescues the anther's dehiscence defect (Kanno et al. 2016). In rice, OsSWEET3a acts as both a sugar transporter and a GA transporter, playing roles in seed germination and early shoot development (Morii et al. 2020).

### GA biosensors and markers

Studies utilizing GA biosensors have provided insights into the transport and localization of GAs, indicating that GAs are highly mobile (Rizza and Jones 2019). Specifically, analysis using the GA perception biosensor GA Perception Sensor 1 (GPS1) revealed that, in the root, the concentration of bioactive GA is highest in the root elongation zone (Rizza et al. 2017). GPS1 represents a pioneering biosensor that utilizes Förster resonance energy transfer (FRET) to detect and track cellular GA levels in planta. This biosensor contains AtGID1C and the N-terminal domain of AtGAI, which are linked to two fluorescent proteins that produce FRET when GA binding to GID1C triggers an intramolecular conformational change. The assessment of a fluorescence emission ratio of nuclear localized-GPS1 (nlsGPS1) enables the precise mapping of endogenous and externally administered GA gradients within various tissue structures at the cellular level. The use of nlsGPS1 live imaging, combined with comprehensive modeling, revealed that a disparity in GA biosynthesis along the roots is accountable for shaping the distribution of GA (Rizza et al. 2021). Another biosensor based on the DELLA protein RGA, named qmRGA (*pRPS5a::RGAmPFYR-VENUS*), provided in planta information on changes in GA responses at the cellular level in the shoot apical meristem, with GA signaling found primarily in cells located between organ primordia (Shi et al. 2021). Furthermore, experiments involving fluorescently labeled bioactive GAs demonstrated the exclusive accumulation of GAs in the root elongation zone (Shani et al. 2013) and in leaf mesophyll cells (Matias-Hernandez et al. 2016), suggesting that GAs move from one tissue to another. The latter process is regulated by two transcription factors, TEMPRANILLO1 (TEM1) and TEM2, which negatively regulate the expression of genes encoding specific GA transporters (GLUCOSINOLATE TRANSPORTER1 (GTR1), NPF3, and NPF2.3) belonging to the NPF family, leading to variable GA accumulation and distribution in mesophyll cells that regulate trichome initiation in the epidermis (Matias-Hernandez et al. 2016).

While the GPS1 FRET biosensor (Rizza et al. 2021) and the qmRGA ratiometric GA signaling biosensor (Shi et al. 2021) report on GA localization based on perception mechanisms, limited progress has been made in generating GA biosensors that are based on transcriptional reporters. Such transcriptional reporters have been widely used in other phytohormone research based on promoters of endogenous phytohormone-induced genes or synthetic transcriptional reporters (Ulmasov et al. 1997; Muller and Sheen 2008; Kim et al. 2011; Okamoto et al. 2013; Liao et al. 2015; Wu et al. 2018). Dayan et al. generated several β-glucuronidase reporters based on the GA-induced promoters of *EXP1*, *MYB34,* and *GA2OX2* and a synthetic GA-responsive promoter (FK) containing known GA-response *cis*-elements found in promoters of α-amylase genes from cereal crops (Dayan et al. 2012). However, constructing a universal GA reporter that reflects the broad range of transcriptional regulation (Fig. 3), remains challenging. Such a reporter would need to respond specifically to endogenous GA levels in all tissues and cell types, with an appropriate reporter turnover rate. It may be difficult to design GA reporters that respond to both DELLA-dependent and -independent pathways.

Despite the progress made, several open questions remain regarding GA transport (see Outstanding questions). One unanswered question is whether there are GA exporters capable of transporting GAs from the cytosol to the apoplast. Currently, no proteins with this function have been identified, but it is believed that such proteins must exist to overcome the GA ion-trapping mechanism. In addition, considering the recent findings regarding GA accumulation in the pericycle vacuole, one could speculate that GA is actively transported out of the vacuole and pericycle cells to reach the endodermis. However, the specific transporters responsible for this process have not yet been identified. Furthermore, the relevance of movement of GA through plasmodesmata and the apoplast remains unclear. A recent discovery demonstrated that the plant hormone ABA travels radially through the plasmodesmata in the root to regulate lateral root branching in response to water stress (air gaps in the soil) (Mehra et al. 2022). Investigating whether a similar mechanism applies to GA in various developmental and environmental responses would be intriguing. Further research is required to fully understand the transport and localization of GAs in plants.

## Concluding remarks

In the last three decades, substantial progress has been made in elucidating the regulation of GA metabolism and the molecular mechanism of GA perception and early GA signaling. The central role of DELLAs as integrators of multiple signaling pathways has clearly been demonstrated, although the specificity of these key growth regulators in distinct tissue/cell-type requires further investigation (see Outstanding Questions). Recently identified GA transporters and the development of GA biosensors are important advances toward understanding how GA regulates plant growth and development in response to internal and external cues (see Outstanding Questions). The development of methods for analyzing GA content at the single-cell level in combination with existing molecular/genomics/proteomics tools will allow us to achieve this goal in the future.

**Outstanding questions**

- How are GA metabolism, transport, and signaling activities regulated in different cell/tissue types to coordinate plant growth and development?
- What is the role of post-transcriptional regulation of GA-metabolic enzymes in determining GA concentration?
- How do DELLAs interact with a myriad of distinct classes of TFs/transcriptional regulators (TRs), and does the tissue/cell-type specific expression of DELLAs and their interacting TFs/TRs determine the selective regulation of a subset of target genes?
- Do GA exporters exist and, if so, what are their developmental roles and specificity?

## Data Availability

No new data were generated in this review.

## Dive Curated Terms

The following phenotypic, genotypic, and functional terms are of significance to the work described in this paper:

SCL3 Gramene: AT1G50420
SCL3 Araport: AT1G50420

GPS1 Gramene: AT2G34630
GPS1 Araport: AT2G34630

ABI5 Gramene: At2g36270
ABI5 Araport: At2g36270

PIF3 Gramene: AT1G09530
PIF3 Araport: AT1G09530

PIF4 Gramene: AT2G43010
PIF4 Araport: AT2G43010

BZR1 Gramene: AT1G75080
BZR1 Araport: AT1G75080

OTS2 Gramene: AT1G10570
OTS2 Araport: AT1G10570

ABI3 Gramene: AT3G24650
ABI3 Araport: AT3G24650

ALC Gramene: AT5G67110
ALC Araport: AT5G67110

MYB23 Gramene: AT5G40330
MYB23 Araport: AT5G40330

GAI Gramene: AT1G14920
GAI Araport: AT1G14920

GID1a Gramene: AT3G05120
GID1a Araport: AT3G05120

GA3ox1 Gramene: At1g15550
GA3ox1 Araport: At1g15550

SPY Gramene: AT3G11540
SPY Araport: AT3G11540

GID1b Gramene: AT3G63010
GID1b Araport: AT3G63010

GID1c Gramene: AT5G27320
GID1c Araport: AT5G27320

RGL1 Gramene: AT1G66350
RGL1 Araport: AT1G66350

RGL2 Gramene: AT3G03450
RGL2 Araport: AT3G03450

BBX24 Gramene: At1g06040
BBX24 Araport: At1g06040

RGA Gramene: At2g01570
RGA Araport: At2g01570

## References

[kiae044-B1] Aach H , Böse G, Graebe JE. *ent*-Kaurene biosynthesis in a cell-free system from wheat (*Triticum aestivum* L.) seedlings and the localization of *ent*-kaurene synthetase in plastids of 3 species. Planta. 1995:197(2):333–342. 10.1007/BF00202655

[kiae044-B2] An F , Zhang X, Zhu Z, Ji Y, He W, Jiang Z, Li M, Guo H. Coordinated regulation of apical hook development by gibberellins and ethylene in etiolated Arabidopsis seedlings. Cell Res. 2012:22(5):915–927. 10.1038/cr.2012.29 22349459 PMC3343656

[kiae044-B3] Anfang M , Shani E. Transport mechanisms of plant hormones. Curr Opin Plant Biol. 2021:63:102055. 10.1016/j.pbi.2021.102055 34102450 PMC7615258

[kiae044-B4] Arana MV , Marin-de la Rosa N, Maloof JN, Blázquez MA, Alabadí D. Circadian oscillation of gibberellin signaling in Arabidopsis. Proc Natl Acad Sci U S A. 2011:108(22):9292–9297. 10.1073/pnas.1101050108 21576475 PMC3107313

[kiae044-B5] Arnaud N , Girin T, Sorefan K, Fuentes S, Wood TA, Lawrenson T, Sablowski R, Østergaard L. Gibberellins control fruit patterning in *Arabidopsis thaliana*. Genes Dev. 2010:24(19):2127–2132. 10.1101/gad.593410 20889713 PMC2947765

[kiae044-B6] Bai MY , Shang JX, Oh E, Fan M, Bai Y, Zentella R, Sun TP, Wang Z-Y. Brassinosteroid, gibberellin, and phytochrome signalling pathways impinge on a common transcription module in Arabidopsis. Nat Cell Biol. 2012:14(8):810–817. 10.1038/ncb2546 22820377 PMC3606816

[kiae044-B7] Barker R , Fernandez Garcia MN, Powers SJ, Vaughan S, Bennett MJ, Phillips AL, Thomas SG, Hedden P. Mapping sites of gibberellin biosynthesis in the Arabidopsis root tip. New Phytol. 2021:229(3):1521–1534. 10.1111/nph.16967 32989730

[kiae044-B8] Bearder JR , MacMillan J, Phinney BO. Fungal products. Part XIV. Metabolic pathways from *ent*-kaurenoic acid to fungal gibberellins in mutant B1-41a of *Gibberella fujikuroi*. J Chem Soc, Perkin Trans. 1975:1(8):721–726. 10.1039/p19750000721

[kiae044-B9] Bensen RJ , Johal GS, Crane VC, Tossberg JT, Schnable PS, Meeley RB, Briggs SP. Cloning and characterization of the maize *AN1* gene. Plant Cell. 1995:7(1):75–84. 10.1105/tpc.7.1.75 7696880 PMC160766

[kiae044-B10] Bhattacharya A , Kourmpetli S, Ward DA, Thomas SG, Gong F, Powers SJ, Carrera E, Taylor B, Gonzalez FND, Tudzynski B, et al Characterization of the fungal gibberellin desaturase as a 2-oxoglutarate-dependent dioxygenase and its utilization for enhancing plant growth. Plant Physiol. 2012:160(2):837–845. 10.1104/pp.112.201756 22911627 PMC3461559

[kiae044-B11] Binenbaum J , Weinstain R, Shani E. Gibberellin localization and transport in plants. Trends Plant Sci. 2018:23(5):410–421. 10.1016/j.tplants.2018.02.005 29530380

[kiae044-B12] Binenbaum J , Wulff N, Camut L, Kiradjiev K, Anfang M, Tal I, Vasuki H, Zhang Y, Sakvarelidze-Achard L, Davière J-M **, et al**. Gibberellin and abscisic acid transporters facilitate endodermal suberin formation in Arabidopsis. Nat Plants. 2023:9:785–802. 10.1038/s41477-023-01391-3 37024660 PMC7615257

[kiae044-B13] Binks R , MacMillan J, Pryce RJ. Plant hormones—VIII: combined gas chromatography-mass spectrometry of methyl esters of gibberellins A_1_ to A_24_ and their trimethylsilyl ethers. Phytochemistry. 1969:8(1):271–284. 10.1016/S0031-9422(00)85825-2

[kiae044-B14] Birch AJ , Rickards RW, Smith H. The biosynthesis of gibberellic acid. Proc Chem Soc. 1958:192–193.

[kiae044-B15] Björklund S , Antti H, Uddestrand I, Moritz T, Sundberg B. Cross-talk between gibberellin and auxin in development of Populus wood: gibberellin stimulates polar auxin transport and has a common transcriptome with auxin. Plant J. 2007:52(3):499–511. 10.1111/j.1365-313X.2007.03250.x 17825053

[kiae044-B16] Blanco-Touriñán N , Legris M, Minguet EG, Costigliolo-Rojas C, Nohales MA, Iniesto E, García-León M, Pacín M, Heucken N, Bloemeir T, et al COP1 destabilizes DELLA proteins in *Arabidopsis*. Proc Natl Acad Sci U S A. 2020a:117(24):13792–13799. 10.1073/pnas.1907969117 32471952 PMC7306988

[kiae044-B17] Blanco-Touriñán N , Serrano-Mislata A, Alabadí D. Regulation of DELLA proteins by post-translational modifications. Plant Cell Physiol. 2020b:61(11):1891–1901. 10.1093/pcp/pcaa113 32886774 PMC7758031

[kiae044-B18] Börner A , Plaschke J, Korzun V, Worland AJ. The relationships between the dwarfing genes of wheat and rye. Euphytica. 1996:89(1):69–75. 10.1007/BF00015721

[kiae044-B19] Bouré N , Arnaud N. Molecular GA pathways as conserved integrators for adaptive responses. Plant Biol. 2023:25(5):649–660. 10.1111/plb.13549 37279043

[kiae044-B20] Brian PW . The effects of some microbial metabolic products on plant growth. Symp Soc Exp Biol. 1957:11:166–181. 13486469

[kiae044-B21] Brian PW , Elson GW, Hemming HG, Radley M. The plant growth promoting properties of gibberellic acid, a metabolic product of the fungus, *Gibberella fujikuroi*. J Sci Food Agric. 1954:5(12):602–612. 10.1002/jsfa.2740051210

[kiae044-B22] Campanaro A , Battaglia R, Galbiati M, Sadanandom A, Tonelli C, Conti L. SUMO proteases OTS1 and 2 control filament elongation through a DELLA-dependent mechanism. Plant Reprod. 2016:29(4):287–290. 10.1007/s00497-016-0292-8 27761651

[kiae044-B23] Camut L , Gallova B, Jilli L, Sirlin-Josserand M, Carrera E, Sakvarelidze-Achard L, Ruffel S, Krouk G, Thomas SG, Hedden P, et al Nitrate signaling promotes plant growth by upregulating gibberellin biosynthesis and destabilization of DELLA proteins. Curr Biol. 2021:31(22):4971–4982.e4974. 10.1016/j.cub.2021.09.024 34614391

[kiae044-B24] Camut L , Regnault T, Sirlin-Josserand M, Sakvarelidze-Achard L, Carrera E, Zumsteg J, Heintz D, Leonhardt N, Lange MJP, Lange T. Root-derived GA_12_ contributes to temperature-induced shoot growth in Arabidopsis. Nat Plants. 2019:5(12):1216–1221. 10.1038/s41477-019-0568-8 31819220

[kiae044-B25] Chandler PM , Marion-Poll A, Ellis M, Gubler F. Mutants at the *Slender1* locus of barley cv Himalaya: molecular and physiological characterization. Plant Physiol. 2002:129(1):181–190. 10.1104/pp.010917 12011349 PMC155882

[kiae044-B26] Chiang HH , Hwang I, Goodman HM. Isolation of the *Arabidopsis GA4* locus. Plant Cell. 1995:7(2):195–201. 10.1105/tpc.7.2.195 7756830 PMC160775

[kiae044-B27] Chiba Y , Shimizu T, Miyakawa S, Kanno Y, Koshiba T, Kamiya Y, Seo M. Identification of *Arabidopsis thaliana* NRT1/PTR family (NPF) proteins capable of transporting plant hormones. J Plant Res. 2015:128(4):679–686. 10.1007/s10265-015-0710-2 25801271

[kiae044-B28] Chin TY , Lockhart JA. Translocation of applied gibberellin in bean seedlings. Am J Bot. 1965:52(8):828–833. 10.1002/j.1537-2197.1965.tb07254.x

[kiae044-B29] Colebrook EH , Thomas SG, Phillips AL, Hedden P. The role of gibberellin signalling in plant responses to abiotic stress. J Exp Biol. 2014:217(1):67–75. 10.1242/jeb.089938 24353205

[kiae044-B30] Conti L , Nelis S, Zhang C, Woodcock A, Swarup R, Galbiati M, Tonelli C, Napier R, Hedden P, Bennett M, et al Small ubiquitin-like modifier protein SUMO enables plants to control growth independently of the phytohormone gibberellin. Dev Cell. 2014:28(1):102–110. 10.1016/j.devcel.2013.12.004 24434138

[kiae044-B31] Cowling RJ , Kamiya Y, Seto H, Harberd NP. Gibberellin dose-response regulation of *GA4* gene transcript levels in Arabidopsis. Plant Physiol. 1998:117(4):1195–1203. 10.1104/pp.117.4.1195 9701576 PMC34884

[kiae044-B32] Crocco CD , Locascio A, Escudero CM, Alabadí D, Blázquez MA, Botto JF. The transcriptional regulator BBX24 impairs DELLA activity to promote shade avoidance in Arabidopsis thaliana. Nat Commun. 2015:6(1):6202. 10.1038/ncomms7202 25656233

[kiae044-B33] Cross BE , Hanson JR, Galt RHB. The biosynthesis of the gibberellins. Part I. (-)-kaurene as a precursor of gibberellic acid. J Chem Soc. 1964:295–300. 10.1039/JR9640000295

[kiae044-B34] Curtis PJ , Cross BE. Gibberellic acid—a new metabolite from the culture filtrates of *Gibberella fujikuroi*. Chem Ind. 1954:1066.

[kiae044-B35] Dai C , Xue HW. Rice early flowering1, a CKI, phosphorylates DELLA protein SLR1 to negatively regulate gibberellin signalling. EMBO J. 2010:29(11):1916–1927. 10.1038/emboj.2010.75 20400938 PMC2885930

[kiae044-B36] David LC , Berquin P, Kanno Y, Seo M, Daniel-Vedele F, Ferrario-Méry S. N availability modulates the role of NPF3.1, a gibberellin transporter, in GA-mediated phenotypes in Arabidopsis. Planta. 2016:244(6):1315–1328. 10.1007/s00425-016-2588-1 27541496

[kiae044-B37] Davière JM , Achard P. A pivotal role of DELLAs in regulating multiple hormone signals. Mol Plant. 2016:9(1):10–20. 10.1016/j.molp.2015.09.011 26415696

[kiae044-B38] Davière JM , Wild M, Regnault T, Baumberger N, Eisler H, Genschik P, Achard P. Class I TCP-DELLA interactions in inflorescence shoot apex determine plant height. Curr Biol. 2014:24(16):1923–1928. 10.1016/j.cub.2014.07.012 25127215

[kiae044-B39] Dayan J , Voronin N, Gong F, Sun TP, Hedden P, Fromm H, Aloni R. Leaf-induced gibberellin signaling is essential for internode elongation, cambial activity, and fiber differentiation in tobacco stems. Plant Cell. 2012:24(1):66–79. 10.1105/tpc.111.093096 22253226 PMC3289570

[kiae044-B40] de Lucas M , Daviere JM, Rodríguez-Falcón M, Pontin M, Iglesias-Pedraz JM, Lorrain S, Fankhauser C, Blázquez MA, Titarenko E, Prat S. A molecular framework for light and gibberellin control of cell elongation. Nature. 2008:451(7177):480–484. 10.1038/nature06520 18216857

[kiae044-B41] Dennis DT , West CA. Biosynthesis of gibberellins. III. Conversion of (-)-kaurene to (-)-kauren-19-oic acid in endosperm of *Echinocystis macrocarpa* greene. J Biol Chem. 1967:242(14):3293–3300. 10.1016/S0021-9258(18)95909-0 4382094

[kiae044-B42] Dill A , Jung H-S, Sun TP. The DELLA motif is essential for gibberellin-induced degradation of RGA. Proc Natl Acad Sci U S A. 2001:98(24):14162–14167. 10.1073/pnas.251534098 11717468 PMC61185

[kiae044-B43] Dill A , Thomas SG, Hu J, Steber CM, Sun TP. The Arabidopsis F-box protein SLEEPY1 targets GA signaling repressors for GA-induced degradation. Plant Cell. 2004:16(6):1392–1405. 10.1105/tpc.020958 15155881 PMC490034

[kiae044-B44] Djakovic-Petrovic T , de Wit M, Voesenek LA, Pierik R. DELLA protein function in growth responses to canopy signals. Plant J. 2007:51(1):117–126. 10.1111/j.1365-313X.2007.03122.x 17488236

[kiae044-B45] Duncan JD , West CA. Properties of kaurene synthetase from *Marah macrocarpus* endosperm—evidence for the participation of separate but interacting enzymes. Plant Physiol. 1981:68(5):1128–1134. 10.1104/pp.68.5.1128 16662063 PMC426057

[kiae044-B46] Evans R , Hanson JR. Studies in terpenoid biosynthesis. Part XIII. Biosynthetic relationship of gibberellins in *Gibberella fujikuroi*. J Chem Soc Perkin Trans. 1975:1(7):663–666. 10.1039/p19750000663

[kiae044-B47] Fall RR , West CA. Purification and properties of kaurene synthetase from *Fusarium moniliforme*. J Biol Chem. 1971:246(22):6913–6928. 10.1016/S0021-9258(19)45933-4 4331199

[kiae044-B48] Feng S , Martinez C, Gusmaroli G, Wang Y, Zhou J, Wang F, Chen L, Yu L, Iglesias-Pedraz JM, Kircher S, et al Coordinated regulation of *Arabidopsis thaliana* development by light and gibberellins. Nature. 2008:451(7177):475–479. 10.1038/nature06448 18216856 PMC2562044

[kiae044-B49] Fonouni-Farde C , Tan S, Baudin M, Brault M, Wen J, Mysore KS, Niebel A, Frugier F, Diet A. DELLA-mediated gibberellin signalling regulates nod factor signalling and rhizobial infection. Nat Commun. 2016:7(1):12636. 10.1038/ncomms12636 27586842 PMC5025792

[kiae044-B50] Foster CA . Slender: an accelerated extension growth mutant of barley. Barley Genet Newsl. 1977:7:24–27.

[kiae044-B51] Fuentes S , Ljung K, Sorefan K, Alvey E, Harberd NP, Østergaard L. Fruit growth in Arabidopsis occurs via DELLA-dependent and DELLA-independent gibberellin responses. Plant Cell. 2012:24(10):3982–3996. 10.1105/tpc.112.103192 23064323 PMC3517231

[kiae044-B52] Fukazawa J , Teramura H, Murakoshi S, Nasuno K, Nishida N, Ito T, Yoshida M, Kamiya Y, Yamaguchi S, Takahashi Y. DELLAs function as coactivators of GAI-ASSOCIATED FACTOR1 in regulation of gibberellin homeostasis and signaling in Arabidopsis. Plant Cell. 2014:26(7):2920–2938. 10.1105/tpc.114.125690 25035403 PMC4145123

[kiae044-B53] Gallego-Bartolomé J , Minguet EG, Grau-Enguix F, Abbas M, Locascio A, Thomas SG, Alabadí D, Blázquez MA. Molecular mechanism for the interaction between gibberellin and brassinosteroid signaling pathways in Arabidopsis. Proc Natl Acad Sci U S A. 2012:109(33):13446–13451. 10.1073/pnas.1119992109 22847438 PMC3421204

[kiae044-B54] Goff SA , Ricke D, Lan TH, Presting G, Wang R, Dunn M, Glazebrook J, Sessions A, Oeller P, Varma H, et al A draft sequence of the rice genome (*Oryza sativa* L. ssp. *japonica*). Science. 2002:296(5565):92–100. 10.1126/science.1068275 11935018

[kiae044-B55] Gomez MD , Ventimilla D, Sacristan R, Perez-Amador MA. Gibberellins regulate ovule integument development by interfering with the transcription factor ATS. Plant Physiol. 2016:172(4):2403–2415. 10.1104/pp.16.01231 27794102 PMC5129715

[kiae044-B56] Graebe JE , Bowen DH, MacMillan J. Conversion of mevalonic acid into gibberellin A_12_-aldehyde in a cell-free system from *Cucurbita pepo*. Planta. 1972:102(3):261–271. 10.1007/BF00386896 24482208

[kiae044-B57] Graebe JE , Dennis DT, Upper CD, West CA. Biosynthesis of gibberellins. I. Biosynthesis of (-)-kaurene, (-)-kauren-19-ol and *trans*-geranylgeraniol in endosperm nucellus of *Echinocystis macrocarpa* greene. J Biol Chem. 1965:240(4):1847–1854. 10.1016/S0021-9258(18)97516-2 14289357

[kiae044-B58] Graebe JE , Hedden P, Gaskin P, MacMillan J. Biosynthesis of a C_19_-gibberellin from mevalonic acid in a cell-free system from a higher plant. Planta. 1974a:120(3):307–309. 10.1007/BF00390299 24442706

[kiae044-B59] Graebe JE , Hedden P, Gaskin P, MacMillan J. Biosynthesis of gibberellins A_12_, A_15_, A_24_, A_36_ and A_37_ by a cell-free system from *Cucurbita maxima*. Phytochem. 1974b:13(8):1433–1440. 10.1016/0031-9422(74)80304-3

[kiae044-B60] Graebe JE , Hedden P, Rademacher W. Gibberellin biosynthesis. In: Lenton JR, editors. Gibberellins—chemistry, physiology and use, vol 5. Wantage: British Plant Growth Regulator Group; 1980. p. 31–47.

[kiae044-B61] Griffiths J , Murase K, Rieu I, Zentella R, Zhang ZL, Powers SJ, Gong F, Phillips AL, Hedden P, Sun TP, et al Genetic characterization and functional analysis of the GID1 gibberellin receptors in Arabidopsis. Plant Cell. 2006:18(12):3399–3414. 10.1105/tpc.106.047415 17194763 PMC1785415

[kiae044-B62] Grove JF . The gibberellins. Q Rev. 1961:15(1):56–70. 10.1039/qr9611500056

[kiae044-B63] Hart GW . Nutrient regulation of signaling and transcription. J Biol Chem. 2019:294(7):2211–2231. 10.1074/jbc.AW119.003226 30626734 PMC6378989

[kiae044-B64] Hasson EP , West CA. Properties of the system for the mixed-function oxidation of kaurene and kaurene derivatives in microsomes of the immature seed of *Marah macrocarpus*: cofactor requirements. Plant Physiol. 1976:58(4):473–478. 10.1104/pp.58.4.473 16659700 PMC543245

[kiae044-B65] Hayashi T . Biochemical studies on ‘bakanae’ fungus of the rice. Part VI. Effect of gibberellin on the activity of amylase in germinated cereal grains. J Agri Chem Soc Japan. 1940:16:531–538.

[kiae044-B66] He J , Chen QW, Xin PY, Yuan J, Ma YH, Wang XM, Xu MM, Chu JF, Peters RJ, Wang GD. CYP72A enzymes catalyse 13-hydrolyzation of gibberellins. Nat Plants. 2019:5(10):1057–1065. 10.1038/s41477-019-0511-z 31527846 PMC7194175

[kiae044-B67] Hedden P . The current status of research on gibberellin biosynthesis. Plant Cell Physiol. 2020:61(11):1832–1849. 10.1093/pcp/pcaa092 32652020 PMC7758035

[kiae044-B68] Hedden P , Croker SJ. Regulation of gibberellin biosynthesis in maize seedlings. In: Karssen CM, Van Loon LC, Vreugdenhil D, editors. Progress in plant growth regulation: proceedings of the 14th international conference on plant growth substances. Dordrecht: Kluwer; 1992. p. 534–544.

[kiae044-B69] Hedden P , Graebe JE. Cofactor requirements for the soluble oxidases in the metabolism of C_20_-gibberellins. J Plant Growth Regul. 1982:1:105–116.

[kiae044-B70] Hedden P , Phillips AL, Rojas MC, Carrera E, Tudzynski B. Gibberellin biosynthesis in plants and fungi: a case of convergent evolution? J Plant Growth Regul. 2001:20(4):319–331. 10.1007/s003440010037 11986758

[kiae044-B71] Hedden P , Phinney BO. Comparison of *ent*-kaurene and *ent*-isokaurene synthesis in cell-free systems from etiolated shoots of normal and *dwarf-5* maize seedlings. Phytochem. 1979:18(9):1475–1479. 10.1016/S0031-9422(00)98478-4

[kiae044-B72] Hedden P , Sponsel V. A century of gibberellin research. J Plant Growth Regul. 2015:34(4):740–760. 10.1007/s00344-015-9546-1 26523085 PMC4622167

[kiae044-B73] Hedden P , Thomas SG. Gibberellin biosynthesis and its regulation. Biochem J. 2012:444(1):11–25. 10.1042/BJ20120245 22533671

[kiae044-B74] Helliwell CA , Chandler PM, Poole A, Dennis ES, Peacock WJ. The CYP88A cytochrome P450, *ent*-kaurenoic acid oxidase, catalyzes three steps of the gibberellin biosynthesis pathway. Proc Natl Acad Sci U S A. 2001a:98(4):2065–2070. 10.1073/pnas.98.4.2065 11172076 PMC29382

[kiae044-B75] Helliwell CA , Sheldon CC, Olive MR, Walker AR, Zeevaart JA, Peacock WJ, Dennis ES. Cloning of the *Arabidopsis ent*-kaurene oxidase gene *GA3*. Proc Natl Acad Sci U S A. 1998:95(15):9019–9024. 10.1073/pnas.95.15.9019 9671797 PMC21195

[kiae044-B76] Helliwell CA , Sullivan JA, Mould RM, Gray JC, Peacock WJ, Dennis ES. A plastid envelope location of *Arabidopsis ent*-kaurene oxidase links the plastid and endoplasmic reticulum steps of the gibberellin biosynthesis pathway. Plant J. 2001b:28(2):201–208. 10.1046/j.1365-313X.2001.01150.x 11722763

[kiae044-B77] Hernández-García J , Briones-Moreno A, Blázquez MA. Origin and evolution of gibberellin signaling and metabolism in plants. Semin Cell Dev Biol. 2021:109:46–54. 10.1016/j.semcdb.2020.04.009 32414681

[kiae044-B78] Hernández-García J , Briones-Moreno A, Dumas R, Blázquez MA. Origin of gibberellin-dependent transcriptional regulation by molecular exploitation of a transactivation domain in DELLA proteins. Mol Biol Evol. 2019:36(5):908–918. 10.1093/molbev/msz009 30668817

[kiae044-B79] Hirano K , Asano K, Tsuji H, Kawamura M, Mori H, Kitano H, Ueguchi-Tanaka M, Matsuoka M. Characterization of the molecular mechanism underlying gibberellin perception complex formation in rice. Plant Cell. 2010:22(8):2680–2696. 10.1105/tpc.110.075549 20716699 PMC2947161

[kiae044-B80] Hoad G , Bowen M. Evidence for gibberellin-like substances in phloem exudate of higher plants. Planta. 1968:82(1):22–32. 10.1007/BF00384695 24519793

[kiae044-B81] Hou X , Hu WW, Shen L, Lee LY, Tao Z, Han JH, Yu H. Global identification of DELLA target genes during Arabidopsis flower development. Plant Physiol. 2008:147(3):1126–1142. 10.1104/pp.108.121301 18502975 PMC2442519

[kiae044-B82] Hou X , Lee LY, Xia K, Yan Y, Yu H. DELLAs modulate jasmonate signaling via competitive binding to JAZs. Dev Cell. 2010:19(6):884–894. 10.1016/j.devcel.2010.10.024 21145503

[kiae044-B83] Hou X , Zhou J, Liu C, Liu L, Shen L, Yu H. Nuclear factor Y-mediated H3K27me3 demethylation of the *SOC1* locus orchestrates flowering responses of Arabidopsis. Nat Commun. 2014:5(1):4601. 10.1038/ncomms5601 25105952

[kiae044-B84] Hu J , Mitchum MG, Barnaby N, Ayele BT, Ogawa M, Nam E, Lai WC, Hanada A, Alonso JM, Ecker JR, et al Potential sites of bioactive gibberellin production during reproductive growth in Arabidopsis. Plant Cell. 2008:20(2):320 336. 10.1105/tpc.107.057752 18310462 PMC2276448

[kiae044-B85] Hu Y , Zhou L, Huang M, He X, Yang Y, Liu X, Li Y, Hou X. Gibberellins play an essential role in late embryogenesis of Arabidopsis. Nat Plants. 2018:4(5):289–298. 10.1038/s41477-018-0143-8 29725104

[kiae044-B86] Hyun Y , Richter R, Vincent C, Martinez-Gallegos R, Porri A, Coupland G. Multi-layered regulation of SPL15 and cooperation with SOC1 integrate endogenous flowering pathways at the Arabidopsis shoot meristem. Dev Cell. 2016:37(3):254–266. 10.1016/j.devcel.2016.04.001 27134142

[kiae044-B87] Ikeda A , Ueguchi-Tanaka M, Sonoda Y, Kitano H, Koshioka M, Futsuhara Y, Matsuoka M, Yamaguchi J. *Slender* rice, a constitutive gibberellin response mutant is caused by a null mutation of the *SLR1* gene, an ortholog of the height-regulating gene *GAI/RGA/RHT/D8*. Plant Cell. 2001:13(5):999–1010. 10.1105/tpc.13.5.999 11340177 PMC135552

[kiae044-B88] Ingram TJ , Reid JB, Murfet IC, Gaskin P, Willis CL, MacMillan J. Internode length in *Pisum*: the *Le* gene controls the 3β-hydroxylation of gibberellin A_20_ to gibberellin A_1_. Planta. 1984:160(5):455–463. 10.1007/BF00429763 24258674

[kiae044-B89] Itoh H , Sasaki A, Ueguchi-Tanaka M, Ishiyama K, Kobayashi M, Hasegawa Y, Minami E, Ashikari M, Matsuoka M. Dissection of the phosphorylation of rice DELLA protein, SLENDER RICE1. Plant Cell Physiol. 2005:46(8):1392–1399. 10.1093/pcp/pci152 15979983

[kiae044-B90] Itoh H , Tanaka-Ueguchi M, Kawaide H, Chen X, Kamiya Y, Matsuoka M. The gene encoding tobacco gibberellin 3β-hydroxylase is expressed at the site of GA action during stem elongation and flower organ development. Plant J. 1999:20(1):15–24. 10.1046/j.1365-313X.1999.00568.x 10571861

[kiae044-B91] Itoh H , Ueguchi-Tanaka M, Sentoku N, Kitano H, Matsuoka M, Kobayashi M. Cloning and functional analysis of two gibberellin 3β-hydroxylase genes that are differently expressed during the growth of rice. Proc Natl Acad Sci U S A. 2001:98(15):8909–8914. 10.1073/pnas.141239398 11438692 PMC37534

[kiae044-B92] Jacobsen SE , Binkowski KA, Olszewski NE. SPINDLY, a tetratricopeptide repeat protein involved in gibberellin signal transduction in *Arabidopsis*. Proc Natl Acad Sci U S A. 1996:93(17):9292–9296. 10.1073/pnas.93.17.9292 8799194 PMC38635

[kiae044-B93] Jacobsen SE , Olszewski NE. Mutations at the *SPINDLY* locus of Arabidopsis alter gibberellin signal transduction. Plant Cell. 1993:5(8):887–896. 10.1105/tpc.5.8.887 8400871 PMC160324

[kiae044-B94] Jasinski S , Tattersall A, Piazza P, Hay A, Martinez-Garcia JF, Schmitz G, Theres K, McCormick S, Tsiantis M. *PROCERA* encodes a DELLA protein that mediates control of dissected leaf form in tomato. Plant J. 2008:56(4):603–612. 10.1111/j.1365-313X.2008.03628.x 18643984

[kiae044-B95] Jin Y , Liu H, Luo D, Yu N, Dong W, Wang C, Zhang X, Dai H, Yang J, Wang E. DELLA proteins are common components of symbiotic rhizobial and mycorrhizal signalling pathways. Nat Commun. 2016:7(1):12433. 10.1038/ncomms12433 27514472 PMC4990646

[kiae044-B96] Josse EM , Gan Y, Bou-Torrent J, Stewart KL, Gilday AD, Jeffree CE, Vaistij FE, Martínez-García JF, Nagy F, Graham IA, et al A DELLA in disguise: SPATULA restrains the growth of the developing Arabidopsis seedling. Plant Cell. 2011:23(4):1337–1351. 10.1105/tpc.110.082594 21478445 PMC3101537

[kiae044-B97] Kamiya Y , Graebe JE. The biosynthesis of all major pea gibberellins in a cell-free system from *Pisum sativum*. Phytochem. 1983:22(3):681–689. 10.1016/S0031-9422(00)86962-9

[kiae044-B98] Kaneko M , Itoh H, Inukai Y, Sakamoto T, Ueguchi-Tanaka M, Ashikari M, Matsuoka M. Where do gibberellin biosynthesis and gibberellin signaling occur in rice plants? Plant J. 2003:35(1):104–115. 10.1046/j.1365-313X.2003.01780.x 12834406

[kiae044-B99] Kanno Y , Oikawa T, Chiba Y, Ishimaru Y, Shimizu T, Sano N, Koshiba T, Kamiya Y, Ueda M, Seo M. AtSWEET13 and AtSWEET14 regulate gibberellin-mediated physiological processes. Nat Commun. 2016:7(1):13245. 10.1038/ncomms13245 27782132 PMC5095183

[kiae044-B100] Kanstrup C , Nour-Eldin HH. The emerging role of the nitrate and peptide transporter family: Npf in plant specialized metabolism. Curr Opin Plant Biol. 2022:68:102243. 10.1016/j.pbi.2022.102243 35709542

[kiae044-B101] Kasahara H , Hanada A, Kuzuyama T, Takagi M, Kamiya Y, Yamaguchi S. Contribution of the mevalonate and methylerythritol phosphate pathways to the biosynthesis of gibberellins in *Arabidopsis*. J Biol Chem. 2002:277(47):45188–45194. 10.1074/jbc.M208659200 12228237

[kiae044-B102] Kim TH , Hauser F, Ha T, Xue S, Böhmer M, Nishimura N, Munemasa S, Hubbard K, Peine N, Lee BH, et al Chemical genetics reveals negative regulation of abscisic acid signaling by a plant immune response pathway. Curr Biol. 2011:21(11):990–997. 10.1016/j.cub.2011.04.045 21620700 PMC3109272

[kiae044-B103] Koornneef M , Elgersma A, Hanhart CJ, van Loenen MEP, van Rijn L, Zeevaart JAD. A gibberellin insensitive mutant of *Arabidopsis thaliana*. Physiol Plant. 1985:65(1):33–39. 10.1111/j.1399-3054.1985.tb02355.x

[kiae044-B104] Koornneef M , van der Veen JH. Induction and analysis of gibberellin sensitive mutants in *Arabidopsis thaliana* (L.) heynh. Theor Appl Genet. 1980:58(6):257–263. 10.1007/BF00265176 24301503

[kiae044-B105] Kurosawa E . Experimental studies on the nature of the substance excreted by the ‘bakanae’ fungus. Transa Nat Hist Soc Formosa. 1926:16:213–227.

[kiae044-B106] Lacombe B , Achard P. Long-distance transport of phytohormones through the plant vascular system. Curr Opin Plant Biol. 2016:34:1–8. 10.1016/j.pbi.2016.06.007 27340874

[kiae044-B107] Lang A . Bolting and flowering in biennial H*yoscyamus niger*, induced by gibberellic acid. Plant Physiol. 1956:31(suppl):35.

[kiae044-B108] Lange T . Purification and partial amino-acid-sequence of gibberellin 20-oxidase from *Cucurbita maxima* L. endosperm. Planta. 1994:195(1):108–115. 10.1007/BF00206298 7765793

[kiae044-B109] Lange T , Hedden P, Graebe JE. Expression cloning of a gibberellin 20-oxidase, a multifunctional enzyme involved in gibberellin biosynthesis. Proc Natl Acad Sci U S A. 1994:91(18):8552–8556. 10.1073/pnas.91.18.8552 8078921 PMC44644

[kiae044-B110] Lange T , Kramer C, Lange MJP. The class III gibberellin 2-oxidases *AtGA2ox9* and *AtGA2ox10* contribute to cold stress tolerance and fertility. Plant Physiol. 2020:184(1):478–486. 10.1104/pp.20.00594 32661062 PMC7479881

[kiae044-B111] Lange MJP , Lange T. Ovary-derived precursor gibberellin A_9_ is essential for female flower development in cucumber. Development. 2016:143(23):4425–4429. 10.1242/dev.135947 27789625

[kiae044-B112] Lantzouni O , Alkofer A, Falter-Braun P, Schwechheimer C. GROWTH-REGULATING FACTORS interact with DELLAs and regulate growth in cold stress. Plant Cell. 2020:32(4):1018–1034. 10.1105/tpc.19.00784 32060178 PMC7145461

[kiae044-B113] Lawit SJ , Wych HM, Xu D, Kundu S, Tomes DT. Maize DELLA proteins dwarf plant8 and dwarf plant9 as modulators of plant development. Plant Cell Physiol. 2010:51(11):1854–1868. 10.1093/pcp/pcq153 20937610

[kiae044-B114] Lee DJ , Zeevaart JAD. Regulation of gibberellin 20-oxidase1 expression in spinach by photoperiod. Planta. 2007:226(1):35–44. 10.1007/s00425-006-0463-1 17216482

[kiae044-B115] Lester DR , Ross JJ, Davies PJ, Reid JB. Mendel’s stem length gene (*Le*) encodes a gibberellin 3 beta-hydroxylase. Plant Cell. 1997:9(8):1435–1443. 10.1105/tpc.9.8.1435 9286112 PMC157009

[kiae044-B116] Lester DR , Ross JJ, Smith JJ, Elliott RC, Reid JB. Gibberellin 2-oxidation and the *SLN* gene of *Pisum sativum*. Plant J. 1999:19(1):65–73. 10.1046/j.1365-313X.1999.00501.x 10417727

[kiae044-B117] Li S , Tian Y, Wu K, Ye Y, Yu J, Zhang J, Liu Q, Hu M, Li H, Tong Y, et al Modulating plant growth-metabolism coordination for sustainable agriculture. Nature. 2018:560(7720):595–600. 10.1038/s41586-018-0415-5 30111841 PMC6155485

[kiae044-B118] Liao CY , Smet W, Brunoud G, Yoshida S, Vernoux T, Weijers D. Reporters for sensitive and quantitative measurement of auxin response. Nat Methods. 2015:12(3):207–210. 10.1038/nmeth.3279 25643149 PMC4344836

[kiae044-B119] Lim S , Park J, Lee N, Jeong J, Toh S, Watanabe A, Kim J, Kang H, Kim DH, Kawakami N, et al ABA-insensitive3, ABA-insensitive5, and DELLAs interact to activate the expression of *SOMNUS* and other high-temperature-inducible genes in imbibed seeds in Arabidopsis. Plant Cell. 2013:25(12):4863–4878. 10.1105/tpc.113.118604 24326588 PMC3903992

[kiae044-B120] Liu Q , Wu K, Song W, Zhong N, Wu Y, Fu X. Improving crop nitrogen use efficiency toward sustainable green revolution. Annu Rev Plant Biol. 2022:73(1):523–551. 10.1146/annurev-arplant-070121-015752 35595292

[kiae044-B121] Locascio A , Blázquez MA, Alabadí D. Dynamic regulation of cortical microtubule organization through prefoldin-DELLA interaction. Curr Biol. 2013:23(9):804–809. 10.1016/j.cub.2013.03.053 23583555

[kiae044-B122] Lovegrove A , Hooley R. Gibberellin and abscisic acid signalling in aleurone. Trends Plant Sci. 2000:5(3):102–110. 10.1016/S1360-1385(00)01571-5 10707075

[kiae044-B123] MacMillan J . Biosynthesis of the gibberellin plant hormones. Nat Prod Rep. 1997:14(3):221–243. 10.1039/np9971400221

[kiae044-B124] Macmillan J , Suter PJ. The occurrence of gibberellin A_1_ in higher plants—isolation from the seed of runner bean (*Phaseolus multiflorus*). Naturwissenschaften. 1958:45(2):46. 10.1007/BF00635028

[kiae044-B125] Magome H , Nomura T, Hanada A, Takeda-Kamiya N, Ohnishi T, Shinma Y, Katsumata T, Kawaide H, Kamiya Y, Yamaguchi S. CYP714B1 and CYP714B2 encode gibberellin 13-oxidases that reduce gibberellin activity in rice. Proc Natl Acad Sci U S A. 2013:110(5):1947–1952. 10.1073/pnas.1215788110 23319637 PMC3562828

[kiae044-B126] Marin-de la Rosa N , Sotillo B, Miskolczi P, Gibbs DJ, Vicente J, Carbonero P, Onate-Sanchez L, Holdsworth MJ, Bhalerao R, Alabadí D, et al Large-scale identification of gibberellin-related transcription factors defines group VII ETHYLENE RESPONSE FACTORS as functional DELLA partners. Plant Physiol. 2014:166(2):1022–1032. 10.1104/pp.114.244723 25118255 PMC4213073

[kiae044-B127] Marín-de la Rosa N , Pfeiffer A, Hill K, Locascio A, Bhalerao RP, Miskolczi P, Grønlund AL, Wanchoo-Kohli A, Thomas SG, Bennett MJ, et al Genome wide binding site analysis reveals transcriptional coactivation of cytokinin-responsive genes by DELLA proteins. PLoS Genet. 2015:11(7):e1005337. 10.1371/journal.pgen.1005337 26134422 PMC4489807

[kiae044-B128] Martin DN , Proebsting WM, Hedden P. Mendel's dwarfing gene: cDNAs from the *Le* alleles and function of the expressed proteins. Proc Natl Acad Sci U S A. 1997:94(16):8907–8911. 10.1073/pnas.94.16.8907 9238076 PMC23192

[kiae044-B129] Martin DN , Proebsting WM, Hedden P. The *SLENDER* gene of pea encodes a gibberellin 2-oxidase. Plant Physiol. 1999:121(3):775–781. 10.1104/pp.121.3.775 10557225 PMC59439

[kiae044-B130] Matías-Hernández L , Aguilar-Jaramillo AE, Osnato M, Weinstain R, Shani E, Suárez-López P, Pelaz S. TEMPRANILLO reveals the mesophyll as crucial for epidermal trichome formation. Plant Physiol. 2016:170(3):1624–1639. 10.1104/pp.15.01309 26802039 PMC4775113

[kiae044-B131] McGinnis KM , Thomas SG, Soule JD, Strader LC, Zale JM, Sun TP, Steber CM. The Arabidopsis *SLEEPY1* gene encodes a putative F-box subunit of an SCF E3 ubiquitin ligase. Plant Cell. 2003:15(5):1120–1130. 10.1105/tpc.010827 12724538 PMC153720

[kiae044-B132] Mehra P , Pandey BK, Melebari D, Banda J, Leftley N, Couvreur V, Rowe J, Anfang M, De Gernier H, Morris E. Hydraulic flux–responsive hormone redistribution determines root branching. Science. 2022:378(6621):762–768. 10.1126/science.add3771 36395221

[kiae044-B133] Mitchum MG , Yamaguchi S, Hanada A, Kuwahara A, Yoshioka Y, Kato T, Tabata S, Kamiya Y, Sun TP. Distinct and overlapping roles of two gibberellin 3-oxidases in Arabidopsis development. Plant J. 2006:45(5):804–818. 10.1111/j.1365-313X.2005.02642.x 16460513

[kiae044-B134] Miyazaki S , Hara M, Ito S, Tanaka K, Asami T, Hayashi K, Kawaide H, Nakajima M. An ancestral gibberellin in a moss *Physcomitrella patens*. Mol Plant. 2018:11(8):1097–1100. 10.1016/j.molp.2018.03.010 29571905

[kiae044-B135] Morii M , Sugihara A, Takehara S, Kanno Y, Kawai K, Hobo T, Hattori M, Yoshimura H, Seo M, Ueguchi-Tanaka M. The dual function of OsSWEET3a as a gibberellin and glucose transporter is important for young shoot development in rice. Plant Cell Physiol. 2020:61(11):1935–1945. 10.1093/pcp/pcaa130 33104219

[kiae044-B136] Morrone D , Chambers J, Lowry L, Kim G, Anterola A, Bender K, Peters RJ. Gibberellin biosynthesis in bacteria: separate *ent*-copalyl diphosphate and *ent*-kaurene synthases in *Bradyrhizobium japonicum*. FEBS Lett. 2009:583(2):475–480. 10.1016/j.febslet.2008.12.052 19121310

[kiae044-B137] Müller B , Sheen J. Cytokinin and auxin interaction in root stem-cell specification during early embryogenesis. Nature. 2008:453(7198):1094–1097. 10.1038/nature06943 18463635 PMC2601652

[kiae044-B138] Murase K , Hirano Y, Sun TP, Hakoshima T. Gibberellin-induced DELLA recognition by the gibberellin receptor GID1. Nature. 2008:456(7221):459–463. 10.1038/nature07519 19037309

[kiae044-B139] Nagel R , Peters RJ. Investigating the phylogenetic range of gibberellin biosynthesis in bacteria. Mol Plant-Microbe Interact. 2017:30(4):343–349. 10.1094/MPMI-01-17-0001-R 28425831 PMC5505637

[kiae044-B140] Nakajima M , Shimada A, Takashi Y, Kim YC, Park SH, Ueguchi-Tanaka M, Suzuki H, Katoh E, Iuchi S, Kobayashi M, et al Identification and characterization of Arabidopsis gibberellin receptors. Plant J. 2006:46(5):880–889. 10.1111/j.1365-313X.2006.02748.x 16709201

[kiae044-B141] Nakamura H , Xue YL, Miyakawa T, Hou F, Qin HM, Fukui K, Shi X, Ito E, Ito S, Park SH, et al Molecular mechanism of strigolactone perception by DWARF14. Nat Commun. 2013:4(1):2613. 10.1038/ncomms3613 24131983

[kiae044-B142] Nett RS , Contreras T, Peters RJ. Characterization of CYP115 as a gibberellin 3-oxidase indicates that certain rhizobia can produce bioactive gibberellin A_4_. ACS Chem Biol. 2017a:12(4):912–917. 10.1021/acschembio.6b01038 28199080 PMC5404427

[kiae044-B143] Nett RS , Montanares M, Marcassa A, Lu X, Nagel R, Charles TC, Hedden P, Rojas MC, Peters RJ. Elucidation of gibberellin biosynthesis in bacteria reveals convergent evolution. Nat Chem Biol. 2017b:13(1):69–74. 10.1038/nchembio.2232 27842068 PMC5193102

[kiae044-B144] Nomura T , Magome H, Hanada A, Takeda-Kamiya N, Mander LN, Kamiya Y, Yamaguchi S. Functional analysis of *Arabidopsis* CYP714a1 and CYP714A2 reveals that they are distinct gibberellin modification enzymes. Plant Cell Physiol. 2013:54(11):1837–1851. 10.1093/pcp/pct125 24009336

[kiae044-B145] Oh E , Zhu JY, Bai MY, Arenhart RA, Sun Y, Wang ZY. Cell elongation is regulated through a central circuit of interacting transcription factors in the Arabidopsis hypocotyl. Elife. 2014:3:e03031. 10.7554/eLife.03031 24867218 PMC4075450

[kiae044-B146] Okada K , Ito T, Fukazawa J, Takahashi Y. Gibberellin induces an increase in cytosolic ca^2+^ via a DELLA-independent signaling pathway. Plant Physiol. 2017:175(4):1536–1542. 10.1104/pp.17.01433 29066668 PMC5717747

[kiae044-B147] Okamoto M , Peterson FC, Defries A, Park SY, Endo A, Nambara E, Volkman BF, Cutler SR. Activation of dimeric ABA receptors elicits guard cell closure, ABA-regulated gene expression, and drought tolerance. Proc Natl Acad Sci U S A. 2013:110(29):12132–12137. 10.1073/pnas.1305919110 23818638 PMC3718107

[kiae044-B148] Olszewski NE , West CM, Sassi SO, Hartweck LM. *O*-GlcNAc protein modification in plants: evolution and function. Biochim Biophys Acta. 2010:1800(2):49–56. 10.1016/j.bbagen.2009.11.016 19961900 PMC2815191

[kiae044-B149] Paleg LG . Physiological effects of gibberellic acid. II. On starch hydrolyzing enzymes of barley endosperm. Plant Physiol. 1960:35(6):902–906. 10.1104/pp.35.6.902 16655440 PMC406057

[kiae044-B150] Park J , Nguyen KT, Park E, Jeon JS, Choi G. DELLA proteins and their interacting RING finger proteins repress gibberellin responses by binding to the promoters of a subset of gibberellin-responsive genes in Arabidopsis. Plant Cell. 2013:25(3):927–943. 10.1105/tpc.112.108951 23482857 PMC3634697

[kiae044-B151] Park J , Oh D, Dassanayaka M, Nguyen KT, Ogas J, Choi G, Sun TP. GA signaling requires chromatin remodeler PICKLE to promote vegetative growth and phase transitions. Plant Physiol. 2017:173(2):1463–1474. 10.1104/pp.16.01471 28057895 PMC5291033

[kiae044-B152] Peng J , Carol P, Richards DE, King KE, Cowling RJ, Murphy GP, Harberd NP. The Arabidopsis *GAI* gene defines a signalling pathway that negatively regulates gibberellin responses. Genes Dev. 1997:11(23):3194–3205. 10.1101/gad.11.23.3194 9389651 PMC316750

[kiae044-B153] Peng J , Harberd NP. Derivative alleles of the Arabidopsis gibberellin-insensitive (*gai*) mutation confer a wild-type phenotype. Plant Cell. 1993:5(3):351–360. 10.2307/3869602 12271067 PMC160276

[kiae044-B154] Peng J , Richards DE, Hartley NM, Murphy GP, Devos KM, Flintham JE, Beales J, Fish LJ, Worland AJ, Pelica F, et al ‘Green revolution’ genes encode mutant gibberellin response modulators. Nature. 1999:400(6741):256–261. 10.1038/22307 10421366

[kiae044-B155] Phillips AL . Genetic control of gibberellin metabolism and signalling in crop improvement. In: Hedden P, Thomas SG, editors. The gibberellins, annual plant reviews, vol 49. Oxford, UK: Wiley Blackwell; 2016. p. 405–430.

[kiae044-B156] Phillips AL , Ward DA, Uknes S, Appleford NEJ, Lange T, Huttly AK, Gaskin P, Graebe JE, Hedden P. Isolation and expression of three gibberellin 20-oxidase cDNA clones from Arabidopsis. Plant Physiol. 1995:108(3):1049–1057. 10.1104/pp.108.3.1049 7630935 PMC157456

[kiae044-B157] Phinney BO . Growth response of single-gene dwarf mutants in maize to gibberellic acid. Proc Natl Acad Sci U S A. 1956:42(4):185–189. 10.1073/pnas.42.4.185 16589846 PMC528248

[kiae044-B158] Phinney BO , Spray C. Chemical genetics and the gibberellin pathway in *Zea mays* L. In: Wareing P, editors. Plant growth substances 1982. New York: Academic Press, London; 1982. p. 101–110.

[kiae044-B159] Pimprikar P , Carbonnel S, Paries M, Katzer K, Klingl V, Bohmer MJ, Karl L, Floss DS, Harrison MJ, Parniske M, et al A CCaMK-CYCLOPS-DELLA complex activates transcription of RAM1 to regulate arbuscule branching. Curr Biol. 2016:26(8):987–998. 10.1016/j.cub.2016.01.069 27020747

[kiae044-B160] Plackett ARG , Powers SJ, Fernandez-Garcia N, Urbanova T, Takebayashi Y, Seo M, Jikumaru Y, Benlloch R, Nilsson O, Ruiz-Rivero O, et al Analysis of the developmental roles of the Arabidopsis gibberellin 20-oxidases demonstrates that GA20ox1, -2, and -3 are the dominant paralogs. Plant Cell. 2012:24(3):941–960. 10.1105/tpc.111.095109 22427334 PMC3336139

[kiae044-B161] Proebsting WM , Hedden P, Lewis MJ, Croker SJ, Proebsting LN. Gibberellin concentration and transport in genetic lines of pea: effects of grafting. Plant Physiol. 1992:100(3):1354–1360. 10.1104/pp.100.3.1354 16653128 PMC1075789

[kiae044-B162] Ptošková K , Szecówka M, Jaworek P, Tarkowská D, Petřík I, Pavlović I, Novák O, Thomas SG, Phillips AL, Hedden P. Changes in the concentrations and transcripts for gibberellins and other hormones in a growing leaf and roots of wheat seedlings in response to water restriction. BMC Plant Biol. 2022:22(1):284. 10.1186/s12870-022-03667-w 35676624 PMC9178827

[kiae044-B163] Pysh LD , Wysocka-Diller JW, Camilleri C, Bouchez D, Benfey PN. The GRAS gene family in Arabidopsis: sequence characterization and basic expression analysis of the *SCARECROW-LIKE* genes. Plant J. 1999:18(1):111–119. 10.1046/j.1365-313X.1999.00431.x 10341448

[kiae044-B164] Qi T , Huang H, Wu D, Yan J, Qi Y, Song S, Xie D. Arabidopsis DELLA and JAZ proteins bind the WD-repeat/bHLH/MYB complex to modulate gibberellin and jasmonate signaling synergy. Plant Cell. 2014:26(3):1118–1133. 10.1105/tpc.113.121731 24659329 PMC4001373

[kiae044-B165] Rademacher W . GROWTH RETARDANTS: effects on gibberellin biosynthesis and other metabolic pathways. Annu Rev Plant Physiol Plant Mol Biol. 2000:51(1):501–531. 10.1146/annurev.arplant.51.1.501 15012200

[kiae044-B166] Rademacher W . Plant growth regulators: backgrounds and uses in plant production. J Plant Growth Regul. 2015:34(4):845–872. 10.1007/s00344-015-9541-6

[kiae044-B167] Ragni L , Nieminen K, Pacheco-Villalobos D, Sibout R, Schwechheimer C, Hardtke CS. Mobile gibberellin directly stimulates Arabidopsis hypocotyl xylem expansion. Plant Cell. 2011:23(4):1322–1336. 10.1105/tpc.111.084020 21498678 PMC3101547

[kiae044-B168] Regnault T , Davière JM, Heintz D, Lange T, Achard P. The gibberellin biosynthetic genes *AtKAO1* and *AtKAO2* have overlapping roles throughout Arabidopsis development. Plant J. 2014:80(3):462–474. 10.1111/tpj.12648 25146977

[kiae044-B169] Regnault T , Davière J-M, Wild M, Sakvarelidze-Achard L, Heintz D, Bergua EC, Diaz IL, Gong F, Hedden P, Achard P. The gibberellin precursor GA_12_ acts as a long-distance growth signal in Arabidopsis. Nat Plants. 2015:1(6):15073. 10.1038/nplants.2015.73 27250008

[kiae044-B170] Reid JB , Ross JJ, Swain SM. Internode length in *Pisum*—a new, slender mutant with elevated levels of C_19_ gibberellins. Planta. 1992:188(4):462–467. 10.1007/BF00197036 24178376

[kiae044-B171] Resentini F , Felipo-Benavent A, Colombo L, Blázquez MA, Alabadí D, Masiero S. TCP14 and TCP15 mediate the promotion of seed germination by gibberellins in *Arabidopsis thaliana*. Mol Plant. 2015:8(3):482–485. 10.1016/j.molp.2014.11.018 25655823

[kiae044-B172] Rieu I , Eriksson S, Powers SJ, Gong F, Griffiths J, Woolley L, Benlloch R, Nilsson O, Thomas SG, Hedden P, et al Genetic analysis reveals that C_19_-GA 2-oxidation is a major gibberellin inactivation pathway in Arabidopsis. Plant Cell. 2008a:20(9):2420–2436. 10.1105/tpc.108.058818 18805991 PMC2570722

[kiae044-B173] Rieu I , Ruiz-Rivero O, Fernandez-Garcia N, Griffiths J, Powers SJ, Gong F, Linhartova T, Eriksson S, Nilsson O, Thomas SG, et al The gibberellin biosynthetic genes *AtGA20ox1* and *AtGA20ox*2 act, partially redundantly, to promote growth and development throughout the Arabidopsis life cycle. Plant J. 2008b:53(3):488–504. 10.1111/j.1365-313X.2007.03356.x 18069939

[kiae044-B174] Rizza A , Jones AM. The makings of a gradient: spatiotemporal distribution of gibberellins in plant development. Curr Opin Plant Biol. 2019:47:9–15. 10.1016/j.pbi.2018.08.001 30173065 PMC6414749

[kiae044-B175] Rizza A , Tang B, Stanley CE, Grossmann G, Owen MR, Band LR, Jones AM. Differential biosynthesis and cellular permeability explain longitudinal gibberellin gradients in growing roots. Proc Natl Acad Sci U S A. 2021:118(8):e1921960118. 10.1073/pnas.1921960118 PMC7923382 33602804

[kiae044-B176] Rizza A , Walia A, Lanquar V, Frommer WB, Jones AM. In vivo gibberellin gradients visualized in rapidly elongating tissues. Nat Plants. 2017:3(10):803–813. 10.1038/s41477-017-0021-9 28970478

[kiae044-B177] Robe K , Barberon M. NPFs rule suberization. Nat Plants. 2023:9(5):689–690. 10.1038/s41477-023-01411-2 37142753

[kiae044-B178] Rojas MC , Hedden P, Gaskin P, Tudzynki B. The P450-1 gene of *Gibberella fujikuro*i encodes a multifunctional enzyme in gibberellin biosynthesis. Proc Natl Acad Sci U S A. 2001:98(10):5838–5843. 10.1073/pnas.091096298 11320210 PMC33300

[kiae044-B179] Ropers HJ , Graebe JE, Gaskin P, Macmillan J. Gibberellin biosynthesis in a cell-free system from immature seeds of *Pisum sativum*. Biochem Biophys Res Commun. 1978:80(4):690–697. 10.1016/0006-291X(78)91299-8 637860

[kiae044-B180] Ross JJ , Reid JB, Swain SM, Hasan O, Poole AT, Hedden P, Willis CL. Genetic-regulation of gibberellin deactivation in *Pisum*. Plant J. 1995:7(3):513–523. 10.1046/j.1365-313X.1995.7030513.x

[kiae044-B181] Saito H , Oikawa T, Hamamoto S, Ishimaru Y, Kanamori-Sato M, Sasaki-Sekimoto Y, Utsumi T, Chen J, Kanno Y, Masuda S. The jasmonate-responsive GTR1 transporter is required for gibberellin-mediated stamen development in Arabidopsis. Nat Commun. 2015:6(1):6095. 10.1038/ncomms7095 25648767 PMC4347201

[kiae044-B182] Sarnowska EA , Rolicka AT, Bucior E, Cwiek P, Tohge T, Fernie AR, Jikumaru Y, Kamiya Y, Franzen R, Schmelzer E, et al DELLA-interacting SWI3C core subunit of switch/sucrose nonfermenting chromatin remodeling complex modulates gibberellin responses and hormonal cross talk in Arabidopsis. Plant Physiol. 2013:163(1):305–317. 10.1104/pp.113.223933 23893173 PMC3762652

[kiae044-B183] Sasaki A , Itoh H, Gomi K, Ueguchi-Tanaka M, Ishiyama K, Kobayashi M, Jeong D-H, An G, Kitano J, Ashikari M, et al Accumulation of phosphorylated repressor for gibberellin signaling in an F-box mutant. Science. 2003:299(5614):1896–1898. 10.1126/science.1081077 12649483

[kiae044-B184] Schomburg FM , Bizzell CM, Lee DJ, Zeevaart JAD, Amasino RM. Overexpression of a novel class of gibberellin 2-oxidases decreases gibberellin levels and creates dwarf plants. Plant Cell. 2003:15(1):151–163. 10.1105/tpc.005975 12509528 PMC143488

[kiae044-B185] Serrano-Mislata A , Bencivenga S, Bush M, Schiessl K, Boden S, Sablowski R. DELLA genes restrict inflorescence meristem function independently of plant height. Nat Plants. 2017:3(9):749–754. 10.1038/s41477-017-0003-y 28827519 PMC5669458

[kiae044-B186] Shani E , Weinstain R, Zhang Y, Castillejo C, Kaiserli E, Chory J, Tsien RY, Estelle M. Gibberellins accumulate in the elongating endodermal cells of Arabidopsis root. Proc Natl Acad Sci U S A. 2013:110(12):4834–4839. 10.1073/pnas.1300436110 23382232 PMC3606980

[kiae044-B187] Shechter I , West CA. Biosynthesis of gibberellins. IV. Biosynthesis of cyclic diterpenes from trans-geranylgeranyl pyrophosphate. J Biol Chem. 1969:244(12):3200–3209. 10.1016/S0021-9258(18)93114-5 4183095

[kiae044-B188] Shi B , Felipo-Benavent A, Cerutti G, Galvan-Ampudia C, Jilli L, Brunoud G, Mutterer J, Vallet E, Sakvarelidze-Achard L, Davière J-M. A quantitative gibberellin signalling biosensor reveals a role for gibberellins in internode specification at the shoot apical meristem. bioRxiv 448154. 10.1101/2021.06.11.448154, 15 June 2021, preprint: not peer reviewed. PMC11079023 38719832

[kiae044-B189] Shimada A , Ueguchi-Tanaka M, Nakatsu T, Nakajima M, Naoe Y, Ohmiya H, Kato H, Matsuoka M. Structural basis for gibberellin recognition by its receptor GID1. Nature. 2008:456(7221):520–523. 10.1038/nature07546 19037316

[kiae044-B190] Silverstone AL , Chang C, Krol E, Sun TP. Developmental regulation of the gibberellin biosynthetic gene *GA1* in *Arabidopsis thaliana*. Plant J. 1997a:12(1):9–19. 10.1046/j.1365-313X.1997.12010009.x 9263448

[kiae044-B191] Silverstone AL , Ciampaglio CN, Sun TP. The Arabidopsis *RGA* gene encodes a transcriptional regulator repressing the gibberellin signal transduction pathway. Plant Cell. 1998:10(2):155–169. 10.1105/tpc.10.2.155 9490740 PMC143987

[kiae044-B192] Silverstone AL , Jung H-S, Dill A, Kawaide H, Kamiya Y, Sun TP. Repressing a repressor: gibberellin-induced rapid reduction of the RGA protein in Arabidopsis. Plant Cell. 2001:13(7):1555–1566. 10.1105/tpc.010047 11449051 PMC139546

[kiae044-B193] Silverstone AL , Mak PYA, Casamitjana Martínez E, Sun TP. The new *RGA* locus encodes a negative regulator of gibberellin response in *Arabidopsis thaliana*. Genetics. 1997b:146(3):1087–1099. 10.1093/genetics/146.3.1087 9215910 PMC1208037

[kiae044-B194] Silverstone AL , Tseng T-S, Swain S, Dill A, Jeong SY, Olszewski NE, Sun TP. Functional analysis of SPINDLY in gibberellin signaling in Arabidopsis. Plant Physiol. 2007:143(2):987–1000. 10.1104/pp.106.091025 17142481 PMC1803720

[kiae044-B195] Simcox PD , Dennis DT, West CA. Kaurene synthetase from plastids of developing plant tissues. Biochem Biophys Res Commun. 1975:66(1):166–172. 10.1016/S0006-291X(75)80309-3 1164423

[kiae044-B196] Sponsel VM . The localization, metabolism and biological activity of gibberellins in maturing and germinating seeds of *Pisum sativum* cv. Progress No. 9. Planta. 1983:159(5):454–468. 10.1007/BF00392082 24258299

[kiae044-B197] Spray C , Phinney BO, Gaskin P, Gilmour SJ, MacMillan J. Internode length in *Zea mays* L.—the *dwarf-1* mutation controls the 3β-hydroxylation of gibberellin A_20_ to gibberellin A_1_. Planta. 1984:160(5):464–468. 10.1007/BF00429764 24258675

[kiae044-B198] Stavang JA , Gallego-Bartolomé J, Gómez MD, Yoshida S, Asami T, Olsen JE, García-Martínez JL, Alabadí D, Blázquez MA. Hormonal regulation of temperature-induced growth in Arabidopsis. Plant J. 2009:60(4):589–601. 10.1111/j.1365-313X.2009.03983.x 19686536

[kiae044-B199] Steber CM , Cooney S, McCourt P. Isolation of the GA-response mutant *sly1* as a suppressor of *ABI1-1* in *Arabidopsis thaliana*. Genetics. 1998:149(2):509–521. 10.1093/genetics/149.2.509 9611170 PMC1460187

[kiae044-B200] Stodola FH , Raper KB, Fennell DI, Conway HF, Sohns VE, Langford CT, Jackson RW. The microbiological production of gibberellins A and X. Arch Biochem Biophys. 1955:54(1):240–245. 10.1016/0003-9861(55)90027-8 13229376

[kiae044-B201] Stowe BB , Yamaki T. The history and physiological action of the gibberellins. Annu Rev Plant Physiol Plant Mol Biol. 1957:8(1):181–216. 10.1146/annurev.pp.08.060157.001145

[kiae044-B202] Sun TP . Gibberellin metabolism, perception and signaling pathways in Arabidopsis. The Arabidopsis Book. 2008:6:e0103. 10.1199/tab.0103 22303234 PMC3243332

[kiae044-B203] Sun TP . The molecular mechanism and evolution of the GA-GID1-DELLA signaling module in plants. Curr Biol. 2011:21(9):R338–R345. 10.1016/j.cub.2011.02.036 21549956

[kiae044-B204] Sun TP . Novel nucleocytoplasmic protein *O*-fucosylation by SPINDLY regulates diverse developmental processes in plants. Curr Opin Struct Biol. 2021:68:113–121. 10.1016/j.sbi.2020.12.013 33476897 PMC8222059

[kiae044-B205] Sun TP , Goodman HM, Ausubel FM. Cloning the Arabidopsis *GA1* locus by genomic subtraction. Plant Cell. 1992:4(2):119–128. 10.2307/3869565 12297643 PMC160113

[kiae044-B206] Sun TP , Gubler F. Molecular mechanism of gibberellin signaling in plants. Annu Rev Plant Biol. 2004:55(1):197–223. 10.1146/annurev.arplant.55.031903.141753 15377219

[kiae044-B207] Sun H , Guo X, Zhu X, Gu P, Zhang W, Tao W, Wang D, Wu Y, Zhao Q, Xu G, et al Strigolactone and gibberellin signaling coordinately regulate metabolic adaptations to changes in nitrogen availability in rice. Mol Plant. 2023:16(3):588–598. 10.1016/j.molp.2023.01.009 36683328

[kiae044-B208] Sun TP , Kamiya Y. The Arabidopsis *GA1* locus encodes the cyclase *ent*-kaurene synthetase A of gibberellin biosynthesis. Plant Cell. 1994:6(10):1509–1518. 10.1105/tpc.6.10.1509 7994182 PMC160538

[kiae044-B209] Takahashi N , Kitamura H, Kawarada A, Seta Y, Takai M, Tamura S, Sumiki Y. Biochemical studies on “Bakanae” fungus. Part XXXIV. Isolation of gibberellins and their properties. Bull Agr Chem Soc Japan. 1955:19:267–277. 10.1080/03758397.1955.10856832

[kiae044-B210] Takehara S , Sakuraba S, Mikama B, Yoshida H, Yoshimura H, Itoh A, Endo M, Watanabe N, Nagae T, Matsuoka M, et al A common allosteric mechanism regulates homeostatic inactivation of auxin and gibberellin. Nat Commun. 2020:11(1):2143. 10.1038/s41467-020-16068-0 32358569 PMC7195466

[kiae044-B211] Tal I , Zhang Y, Jørgensen ME, Pisanty O, Barbosa IC, Zourelidou M, Regnault T, Crocoll C, Olsen CE, Weinstain R, et al The Arabidopsis NPF3 protein is a GA transporter. Nat Commun. 2016:7(1):11486. 10.1038/ncomms11486 27139299 PMC4857387

[kiae044-B212] Tanaka J , Yano K, Aya K, Hirano K, Takehara S, Koketsu E, Ordonio RL, Park S-H, Nakajima M, Ueguchi-Tanaka M. Antheridiogen determines sex in ferns via a spatiotemporally split gibberellin synthesis pathway. Science. 2014:346(6208):469–473. 10.1126/science.1259923 25342803

[kiae044-B213] Thomas SG , Bláquez MA, Alabadí D. DELLA Proteins: master regulators of gibberellin-responsive growth and development. In: Hedden P, Thomas SG, editors. Annual plant reviews, vol 49. Chichester: Wiley; 2016. p. 189–228.

[kiae044-B214] Thomas SG , Phillips AL, Hedden P. Molecular cloning and functional expression of gibberellin 2-oxidases, multifunctional enzymes involved in gibberellin deactivation. Proc Natl Acad Sci U S A. 1999:96(8):4698–4703. 10.1073/pnas.96.8.4698 10200325 PMC16395

[kiae044-B215] Ueguchi-Tanaka M , Ashikari M, Nakajima M, Itoh H, Katoh E, Kobayashi M, Chow TY, Hsing YI, Kitano H, Yamaguchi I, et al *GIBBERELLIN INSENSITIVE DWARF1* encodes a soluble receptor for gibberellin. Nature. 2005:437(7059):693–698. 10.1038/nature04028 16193045

[kiae044-B216] Ulmasov T , Murfett J, Hagen G, Guilfoyle TJ. Aux/IAA proteins repress expression of reporter genes containing natural and highly active synthetic auxin response elements. Plant Cell. 1997:9(11):1963–1971. 10.1105/tpc.9.11.1963 9401121 PMC157050

[kiae044-B217] Van De Velde K , Ruelens P, Geuten K, Rohde A, Van Der Straeten D. Exploiting DELLA signaling in cereals. Trends Plant Sci. 2017:22(10):880–893. 10.1016/j.tplants.2017.07.010 28843766

[kiae044-B218] Van De Velde K , Thomas SG, Heyse F, Kaspar R, Van Der Straeten D, Rohde A. N-terminal truncated RHT-1 proteins generated by translational reinitiation cause semi-dwarfing of wheat green revolution alleles. Mol Plant. 2021:14(4):679–687. 10.1016/j.molp.2021.01.002 33422695

[kiae044-B219] Wang F , Yoshida H, Matsuoka M. Making the ‘green revolution’ truly green: improving crop nitrogen use efficiency. Plant Cell Physiol. 2021:62(6):942–947. 10.1093/pcp/pcab051 33836084

[kiae044-B220] Weiss D , Halevy AH. Stamens and gibberellin in the regulation of corolla pigmentation and growth in *Petunia hybrida*. Planta. 1989:179(1):89–96. 10.1007/BF00395775 24201426

[kiae044-B221] West CA . Biosynthesis of gibberellins. In: Milborrow BV, editors. Biosynthesis and its control in plants. London, New York: Academic Press; 1973. p. 473–482.

[kiae044-B222] Weston DE , Elliott RC, Lester DR, Rameau C, Reid JB, Murfet IC, Ross JJ. The pea DELLA proteins LA and CRY are important regulators of gibberellin synthesis and root growth. Plant Physiol. 2008:147(1):199–205. 10.1104/pp.108.115808 18375599 PMC2330316

[kiae044-B223] Wexler S , Schayek H, Rajendar K, Tal I, Shani E, Meroz Y, Dobrovetsky R, Weinstain R. Characterizing gibberellin flow *in planta* using photocaged gibberellins. Chem Sci. 2019:10(5):1500–1505. 10.1039/C8SC04528C 30809367 PMC6354844

[kiae044-B224] Wild M , Davière JM, Cheminant S, Regnault T, Baumberger N, Heintz D, Baltz R, Genschik P, Achard P. The Arabidopsis DELLA *RGA-LIKE3* is a direct target of MYC2 and modulates jasmonate signaling responses. Plant Cell. 2012:24(8):3307–3319. 10.1105/tpc.112.101428 22892320 PMC3462633

[kiae044-B225] Wild M , Daviere JM, Regnault T, Sakvarelidze-Achard L, Carrera E, Lopez Diaz I, Cayrel A, Dubeaux G, Vert G, Achard P. Tissue-specific regulation of gibberellin signaling fine-tunes Arabidopsis iron-deficiency responses. Dev Cell. 2016:37(2):190–200. 10.1016/j.devcel.2016.03.022 27093087

[kiae044-B226] Williams J , Phillips AL, Gaskin P, Hedden P. Function and substrate specificity of the gibberellin 3β-hydroxylase encoded by the Arabidopsis *GA4* gene. Plant Physiol. 1998:117(2):559–563. 10.1104/pp.117.2.559 9625708 PMC34975

[kiae044-B227] Willige BC , Ghosh S, Nill C, Zourelidou M, Dohmann EMN, Maier A, Schwechheimer C. The DELLA domain of GA INSENSITIVE mediates the interaction with the GA INSENSITIVE DWARF1A gibberellin receptor of Arabidopsis. Plant Cell. 2007:19(4):1209–1220. 10.1105/tpc.107.051441 17416730 PMC1913748

[kiae044-B228] Wittwer SH , Bukovac MJ, Sell HM, Weller LE. Some effects of gibberellin on flowering and fruit setting. Plant Physiol. 1957:32(1):39–41. 10.1104/pp.32.1.39 16654939 PMC540856

[kiae044-B229] Wu R , Duan L, Pruneda-Paz JL, Oh DH, Pound M, Kay S, Dinneny JR. The *6xABRE* synthetic promoter enables the spatiotemporal analysis of ABA-mediated transcriptional regulation. Plant Physiol. 2018:177(4):1650–1665. 10.1104/pp.18.00401 29884679 PMC6084650

[kiae044-B230] Wu K , Wang SS, Song WZ, Zhang JQ, Wang Y, Liu Q, Yu JP, Ye YF, Li S, Chen JF, et al Enhanced sustainable green revolution yield via nitrogen-responsive chromatin modulation in rice. Science. 2020:367(6478):eaaz2046. 10.1126/science.aaz2046 32029600

[kiae044-B231] Wulff N , Ernst HA, Jørgensen ME, Lambertz S, Maierhofer T, Belew ZM, Crocoll C, Motawia MS, Geiger D, Jørgensen FS. An optimized screen reduces the number of GA transporters and provides insights into nitrate transporter 1/peptide transporter family substrate determinants. Front Plant Sci. 2019:10:1106. 10.3389/fpls.2019.01106 31632416 PMC6785635

[kiae044-B232] Xu YL , Li L, Wu KQ, Peeters AJM, Gage DA, Zeevaart JAD. The *GA5* locus of *Arabidopsis thaliana* encodes a multifunctional gibberellin 20-oxidase—molecular cloning and functional expression. Proc Natl Acad Sci U S A. 1995:92(14):6640–6644. 10.1073/pnas.92.14.6640 7604047 PMC41574

[kiae044-B233] Xu F , Li T, Xu PB, Li L, Du SS, Lian HL, Yang HQ. DELLA proteins physically interact with CONSTANS to regulate flowering under long days in Arabidopsis. FEBS Lett. 2016:590(4):541–549. 10.1002/1873-3468.12076 26801684

[kiae044-B234] Yabuta T , Sumiki T. Communication to the editor. J Agr Chem Soc Japan. 1938:14:1526.

[kiae044-B235] Yamaguchi S . Gibberellin metabolism and its regulation. Annu Rev Plant Biol. 2008:59(1):225–251. 10.1146/annurev.arplant.59.032607.092804 18173378

[kiae044-B236] Yamaguchi S , Kamiya Y. Gibberellin biosynthesis: its regulation by endogenous and environmental signals. Plant Cell Physiol. 2000:41(3):251–257. 10.1093/pcp/41.3.251 10805587

[kiae044-B237] Yamaguchi S , Kamiya Y, Sun T. Distinct cell-specific expression patterns of early and late gibberellin biosynthetic genes during Arabidopsis seed germination. Plant J. 2001:28(4):443–453. 10.1046/j.1365-313X.2001.01168.x 11737781

[kiae044-B238] Yamaguchi S , Saito T, Abe H, Yamane H, Murofushi N, Kamiya Y. Molecular cloning and characterization of a cDNA encoding the gibberellin biosynthetic enzyme *ent*-kaurene synthase B from pumpkin (*Cucurbita maxima* L.). Plant J. 1996:10(2):203–213. 10.1046/j.1365-313X.1996.10020203.x 8771778

[kiae044-B239] Yamaguchi S , Sun T, Kawaide H, Kamiya Y. The *GA2* locus of *Arabidopsis thaliana* encodes *ent*-kaurene synthase of gibberellin biosynthesis. Plant Physiol. 1998:116(4):1271–1278. 10.1104/pp.116.4.1271 9536043 PMC35033

[kiae044-B240] Yan J , Li X, Zeng B, Zhong M, Yang J, Yang P, Li X, He C, Lin J, Liu X, et al FKF1 F-box protein promotes flowering in part by negatively regulating DELLA protein stability under long-day photoperiod in Arabidopsis. J Integr Plant Biol. 2020:62(11):1717–1740. 10.1111/jipb.12971 32427421

[kiae044-B241] Yang DL , Yao J, Mei CS, Tong XH, Zeng LJ, Li Q, Xiao LT, Sun TP, Li J, Deng XW, et al Plant hormone jasmonate prioritizes defense over growth by interfering with gibberellin signaling cascade. Proc Natl Acad Sci U S A. 2012:109(19):E1192–E1200. 10.1073/pnas.1201616109 22529386 PMC3358897

[kiae044-B242] Yoshida H , Hirano K, Sato T, Mitsuda N, Nomoto M, Maeo K, Koketsu E, Mitani R, Kawamura M, Ishiguro S, et al DELLA protein functions as a transcriptional activator through the DNA binding of the INDETERMINATE DOMAIN family proteins. Proc Natl Acad Sci U S A. 2014:111(21):7861–7866. 10.1073/pnas.1321669111 24821766 PMC4040565

[kiae044-B243] Yoshida H , Takehara S, Mori M, Ordonio RL, Matsuoka M. Evolution of GA metabolic enzymes in land plants. Plant Cell Physiol. 2020:61(11):1919–1934. 10.1093/pcp/pcaa126 33049049

[kiae044-B244] Yu S , Galvão VC, Zhang YC, Horrer D, Zhang TQ, Hao YH, Feng YQ, Wang S, Schmid M, Wang JW. Gibberellin regulates the Arabidopsis floral transition through miR156-targeted SQUAMOSA promoter binding-like transcription factors. Plant Cell. 2012:24(8):3320–3332. 10.1105/tpc.112.101014 22942378 PMC3462634

[kiae044-B245] Yu J , Hu S, Wang J, Wong GK, Li S, Liu B, Deng Y, Dai L, Zhou Y, Zhang X, et al A draft sequence of the rice genome (*Oryza sativa* L. ssp. *indica*). Science. 2002:296(5565):79–92. 10.1126/science.1068037 11935017

[kiae044-B246] Zentella R , Hu J, Hsieh WP, Matsumoto PA, Dawdy A, Barnhill B, Oldenhof H, Hartweck LM, Maitra S, Thomas SG, et al *O*-GlcNAcylation of master growth repressor DELLA by SECRET AGENT modulates multiple signaling pathways in Arabidopsis. Genes Dev. 2016:30(2):164–176. 10.1101/gad.270587.115 26773002 PMC4719307

[kiae044-B247] Zentella R , Sui N, Barnhill B, Hsieh WP, Hu J, Shabanowitz J, Boyce M, Olszewski NE, Zhou P, Hunt DF, et al The Arabidopsis *O*-fucosyltransferase SPINDLY activates nuclear growth repressor DELLA. Nat Chem Biol. 2017:13(5):479–485. 10.1038/nchembio.2320 28244988 PMC5391292

[kiae044-B248] Zentella R , Zhang ZL, Park M, Thomas SG, Endo A, Murase K, Fleet CM, Jikumaru Y, Nambara E, Kamiya Y, et al Global analysis of DELLA direct targets in early gibberellin signaling in Arabidopsis. Plant Cell. 2007:19(10):3037–3057. 10.1105/tpc.107.054999 17933900 PMC2174696

[kiae044-B249] Zhang Y , Berman A, Shani E. Plant hormone transport and localization: signaling molecules on the move. Annu Rev Plant Biol. 2023:74(1):453–479. 10.1146/annurev-arplant-070722-015329 36889002

[kiae044-B250] Zhang D , Jing Y, Jiang Z, Lin R. The chromatin-remodeling factor PICKLE integrates brassinosteroid and gibberellin signaling during skotomorphogenic growth in Arabidopsis. Plant Cell. 2014:26(6):2472–2485. 10.1105/tpc.113.121848 24920333 PMC4114946

[kiae044-B251] Zhang ZL , Ogawa M, Fleet CM, Zentella R, Hu J, Heo J-O, Lim J, Kamiya Y, Yamaguchi S, Sun TP. SCARECROW-LIKE 3 promotes gibberellin signaling by antagonizing DELLA in Arabidopsis. Proc Natl Acad Sci U S A. 2011:108(5):2160–2165. 10.1073/pnas.1012232108 21245327 PMC3033277

[kiae044-B252] Zhu YY , Nomura T, Xu YH, Zhang YY, Peng Y, Mao BZ, Hanada A, Zhou HC, Wang RX, Li PJ, et al *ELONGATED UPPERMOST INTERNODE* encodes a cytochrome P450 monooxygenase that epoxidizes gibberellins in a novel deactivation reaction in rice. Plant Cell. 2006:18(2):442–456. 10.1105/tpc.105.038455 16399803 PMC1356550

[kiae044-B253] Zweig G , Yamaguchi S, Mason GW. Translocation of C^14^-gibberellin in red kidney bean, normal corn, and dwarf corn. Advances Chem. 1961:28:122–134. 10.1021/ba-1961-0028.ch013

